# Dissociations between stereoacuity and visual acuity with binocular night vision goggles

**DOI:** 10.3758/s13414-026-03285-w

**Published:** 2026-06-22

**Authors:** Mike Tombu

**Affiliations:** Defence Research and Development Canada, Toronto Research Centre, 1133 Sheppard Ave West, Toronto, ON M3K 2C9 Canada

**Keywords:** Stereopsis, Night vision goggles, Depth perception, Binocular disparity, Visual acuity

## Abstract

Night vision goggles (NVGs) enhance vision under scotopic conditions by amplifying near-infrared light and converting it to a wavelength that can be seen by the human visual system. Although significantly more expensive than monocular systems, binocular NVGs provide the user with independent inputs to each eye, and therefore facilitate stereopsis, the ability to discriminate differences in depth from binocular cues. Past work examining stereopsis through NVGs has been mixed, with early-generation systems showing no evidence for stereopsis while more-modern systems show some evidence for (and others against) stereopsis, albeit at levels below that observed under natural photopic viewing conditions. These studies have examined stereopsis through NVGs under ideal conditions that isolate binocular contributions to depth perception. In the present study, stereopsis through NVGs was examined under more realistic conditions and for more operationally relevant tasks. The results show that stereopsis through NVGs persists even under degraded viewing conditions and provides a binocular advantage for real-world tasks where the perception of depth plays an important role. These results show that limitations in stereoacuity through NVGs are not driven by visual acuity limits, as has been previously argued, and more generally a dissociation between visual acuity and stereoacuity.

Conducting military operations at night has traditionally been challenging due to human night vision limitations (United States Army, 1986). Scotopic vision is characterized by reduced spatial acuity, a lack of colour information, and sluggish temporal processing relative to photopic vision (Barbur & Stockman, 2010). These shortcomings significantly limit target detection, recognition and identification distances, and by extension impair situational awareness (Taylor, 1960, as cited in Dyer et al., 1995). To gain an advantage under low-light conditions, considerable investment has been made to develop systems that enhance human night vision, thus permitting the conduct of more effective low-light operations (Wiseman, 1991).

Night vision goggles (NVGs) equipped with image intensification tubes are one of the most ubiquitous night vision enhancement tools in use by today’s militaries. Image intensification tubes use an objective lens to capture visible and near-infrared light from stars, the moon, and other natural and artificial sources. These photons are then converted to electrons by a photocathode before being amplified in a many-to-one fashion by a microchannel plate. The resulting electrons are then projected onto a phosphor screen, which converts them into a visible spectrum image that can be seen by the user (Parush et al., 2011; Task, 1992).

While NVGs dramatically enhance night vision, they do not turn night into day (Verona & Rash, 1989). Relative to unaided photopic vision, visual acuity through NVGs is significantly impaired, with the degree of impairment becoming more pronounced as ambient light levels and target contrast decrease (Higginbotham, 2006; Kotulak & Rash, 1992; Pinkus & Task, 1998; Wiley, 1989). Similarly, the field of view provided by NVGs (typically ~ 40°) is only a fraction of that experienced with unaided binocular vision (210°; Howard & Rogers, 2012), and the monochromatic nature of the image produced by NVGs removes potentially useful colour information. Unlike the eyes, NVGs are equipped with a manual focus, meaning that scene elements off the focal plane will remain blurred, even when fixated. Finally, due to the shifted nodal point of NVGs, objects typically appear 10–15 cm closer through NVGs than they are (Hadani, 1991). Despite these significant deficiencies, NVGs greatly enhance soldier performance in low-light settings (Dyer & Young, 1998; Wiley, 1989).

NVGs can be equipped with an image-intensification tube for each eye, thus providing a binocular input to the user. Although binocular NVGs are typically designed so that the field of view of the two eyes are maximally overlapping and therefore do not increase the field of view of the system, they do provide binocular cues for depth not available in a monocular system. For this reason (as well as the redundancy provided by having two tubes), militaries are prepared to spend two to three times the cost of a monocular system to equip soldiers, sailors and aviators with binocular NVGs.

## Binocular depth cues

Binocular visual information is processed through both comparative and summative streams of processing, both of which can provide valuable information on spatial layout (Harrington et al., 2014).

Comparative stream processes provide powerful cues to 3D layout and are often credited with conveying the sense of ‘real’ depth experienced under natural binocular viewing conditions (Vishwanath & Hibbard, 2013). Because the eyes have slightly different views of the world caused by their physical offset, the visual projection to each one is slightly different. Differences in the projected images are more pronounced for closer elements in the visual scene, which means that binocular disparities, or differences in the spatial position of elements in the visual image as received by each eye, can be used to estimate how far away the elements are.

Similarly, when fixating an object, the angle formed by the optical axes of the two eyes (binocular convergence) will depend on the distance to the fixated object and can therefore provide depth information. For objects at the horizon, this angle will approach zero, increasing as the fixated object becomes less distal. At relatively short distances (< 20 m) both binocular disparities and binocular convergence provide compelling cues to spatial layout (Brenner & Smeets, 2018; Cutting & Vishton, 1995).

Examining summative processes, Jones and Lee (1981) found that binocular concordance (agreement between the information arriving at each eye) enhanced performance for a variety of visuomotor tasks that depend on depth perception, under conditions in which comparative stream information was not available. They had participants perform a task, such as bead threading, while watching their hands on a closed-circuit television. The task was performed both monocularly and binocularly. Despite the absence of comparative cues, performance was better under binocular conditions, thus demonstrating the importance of summative processes. Taken together, binocular vision provides both comparative (binocular disparities, convergence) and summative (binocular concordance) mechanisms supporting the perception of spatial layout.

Binocular depth cues are however not the only cues used to judge depth. Powerful monocular cues such as occlusion, motion perspective, relative size, relative density, height in the visual field, aerial perspective and accommodation also provide information on spatial layout and can be used to provide an accurate representation of space (Cutting & Vishton, 1995). Although Palmisano et al. (2010) have shown evidence that under tightly controlled, visually impoverished viewing, binocular depth cues continue to provide useful depth information for objects out to 250 m, under more natural, visually richer conditions, binocular depth cues are only effective to 20 m (McCann et al., 2018), with monocular cues driving performance at longer distances.

Given these findings, the extent to which binocular NVGs enhance depth perception, and by extension operator performance, has been of interest to militaries since at least the 1970s (see Harrington et al., 2014, for a review) and is the focus of the research reported herein.

## Binocular depth perception through NVGs

Although a binocular advantage for depth perception is well established under natural viewing conditions (e.g., Hayhoe et al., 2009; McKee & Taylor, 2010), past work has found only limited improvement through NVGs. Wiley and Holly (1976; see also Wiley et al., 1976) used a Howard-Dolman apparatus to evaluate stereoacuity (the ability to discriminate differences in depth from binocular cues) through second-generation AN/PVS-5 binocular NVGs. The Howard-Dolman apparatus consists of a box containing two rods that can be observed through a horizontal viewport. One rod is fixed, and the other can be adjusted in depth relative to the viewport. Participants view the rods from a fixed distance (typically 3–6 m; 6 m in the case of Wiley & Holly, 1976) and are required to align the adjustable rod with the fixed rod in depth. The apparatus and associated procedure are designed to minimize monocular depth cues (by fixing the head and presenting the rods at eye level, against a uniform background under diffuse lighting to eliminate shadows), thereby isolating (as much as possible) the binocular contribution to depth perception. The deviation in depth between the two rods (in mm or in terms of angular separation) is used to calculate a depth discrimination threshold. To determine the stereoacuity of the participant, the task is performed both monocularly and binocularly. Participants with good stereoacuity will show a marked improvement in task performance under binocular viewing conditions.

Depth discrimination thresholds should not be considered as an absolute measure of performance, but rather as a relative measure. Different tests will produce different values (Berry et al., 1950), often varying substantially in terms of absolute value. However, relative differences between conditions assessed using the same test (and testing conditions) can be used to judge the quality of depth perception achieved in a given condition. For example, performance in a condition of interest can be compared with unaided binocular performance, unaided monocular performance, or both to establish the extent to which a given condition preserves stereopsis.

Wiley and Holly (1976) had participants perform the Howard-Dolman task monocularly and binocularly under natural, well-lit viewing conditions as well as through NVGs. Under natural viewing a significant binocular advantage was observed, with depth discrimination thresholds improving from 19.3 arcsecs to 5.0 arcsecs. However, no such advantage was observed through NVGs. Depth discrimination thresholds through NVGs were roughly (and statistically) equivalent to monocular viewing under natural conditions whether the task was performed monocularly (26.2 arcsec) or binocularly (17.9 arcsec). Wiley (1989) reported similar results.

Armentrout (1993) examined stereoacuity through third generation binocular NVGs using a procedure similar to that used by Wiley and colleagues. A Howard-Dolman apparatus at 6 m was again used to determine depth discrimination thresholds under both day (unaided) and night (through an AN/PVS-6 binocular NVG) conditions. Both illumination level and the contrast of the rods relative to the background were manipulated. Performance was only assessed binocularly. Replicating Wiley and colleagues, performance was significantly worse under night conditions through the NVG (mean error 74.77 mm collapsing across illumination and contrast conditions) than unaided under day conditions (35.92 mm collapsing across illumination and contrast conditions). Because no monocular conditions were examined, it is unclear whether the binocular NVG condition enhanced depth perception relative to monocular levels, only that stereoacuity performance fell short of unaided day levels. In addition to viewing condition (day vs night), a significant effect of contrast was also observed. Thresholds were significantly higher (12–14%) in the low-contrast condition (25% contrast) than in the mid-contrast and high-contrast conditions (53% and 83% contrast).

The lack of an observable binocular advantage in depth discrimination through NVGs in the early literature may have to do with the quality of the image produced by second and early third generation tubes. Tubes of this period tended to be relatively dim (1–3 cd/m^2^ for second-generation tubes and 2.4–7.5 cd/m^2^ for third generation tubes; Verona & Rash, 1989), produced a relatively low-resolution image (best Snellen visual acuity 20/50 for second generation and 20/40 for third generation; Bender et al., 1992; Dyer & Young, 1998), and suffered from contrast loss (Rabin, 1996).

Each of these factors has been shown to impair stereopsis under natural viewing conditions. Mueller and Lloyd (1948; see also Berry et al., 1950) demonstrated that depth discrimination thresholds increase with decreasing luminance, and Halpern and Blake (1988) showed the negative impact of decreasing contrast. Goodwin and Romano (1985; see also Donzis et al., 1983; Levy & Glick, 1974; Westheimer & McKee, 1980) examined the relationship between visual acuity and stereoacuity by manipulating the visual acuity of normal observers and measuring stereoacuity. They found a precipitous drop in stereoacuity as visual acuity decreased from 20/25 to 20/50 (from 55 arcsecs to ~ 700 arcsecs). The drop was most pronounced when the visual acuity of only one eye was degraded, holding the other at 20/20.

Stereoacuity through better-performing third generation NVGs was tested by Knight et al. (1998), again with the Howard-Dolman apparatus at 6 m, this time using both an ANVIS (AN/AVS-6) NVG and a newer F4949 (AN/AVS-9). An unaided, binocular day condition was used as a baseline and both NVGs were tested binocularly, while the F4949 was also tested monocularly. All NVG testing was conducted at ¾-moon illumination. Most participants were able to achieve 20/35 visual acuity or better with both NVGs at this ambient light level. Although neither binocular NVG condition allowed participants to achieve the level of stereoacuity attainable under binocular day conditions (6.48 arcsec), both exceeded monocular performance with the F4949 (18.42 arcsec for the ANVIS and 17.35 arcsec for the F4949 binocularly compared with 38.83 arcsec for the F4949 monocularly). This result shows that binocular NVGs equipped with more modern I^2^ tubes do provide the user with some stereopsis, albeit at a level still below that to which they are accustom under natural day conditions.

However, not all studies employing more modern third generation binocular NVGs have found a binocular advantage for depth perception. Merritt et al. (2006) captured videos from the left and right tubes of a third generation F4949 NVG of two human targets. The videos essentially depicted a human analog of the Howard-Dolman apparatus. Both targets faced away from the camera, with one stationary, and the other moving towards and then past the stationary target. The participants used polarized ‘3D’ glasses that allowed the researchers to present a different video to each eye. Stereopsis was evaluated by comparing performance in a biocular condition where participants were presented with the same video to both eyes, with a binocular condition in which the video from the left tube was presented to the left eye and the video from the right tube was presented to the right eye. As a result, in the binocular condition binocular disparities (and binocular convergence) were present, providing additional cues to spatial layout. The participants’ task was to indicate when the moving target was shoulder to shoulder with the stationary target. If binocular disparities facilitate depth perception through NVGs, misalignment should be less in the binocular condition than in the biocular condition. However, the results showed that binocular and biocular conditions did not differ, indicating that participants were not able to take advantage of binocular depth cues under these conditions.

CuQlock-Knopp and colleagues (1995, 1996) examined terrain traverse with monocular (AN/AVS-6 with one tube removed), biocular (AN/PVS-7 s), and binocular NVGs (AN/PVS-6) under no-moon and ¾-moon conditions and found that binocular viewing improved speed of traverse under no-moon conditions, but not under ¾-moon conditions. The direction of the dependence between performance and ambient lighting is however not consistent with stereopsis, which typically degrades as both luminance (Berry et al., 1950; Mueller & Lloyd, 1948) and contrast (Halpern & Blake, 1988) degrade. If stereopsis were driving the binocular advantage for terrain traverse, one would expect the size of the advantage to decrease as conditions darken due to reduced contrast and luminance in the image produced by the NVG. However, the opposite pattern of results was observed.

Interestingly, summative processes typically follow the observed pattern of results. Laird (1924) and Shaad (1935) have both shown that binocular summation (improved binocular performance compared with monocular performance on a visual task; Blake & Fox, 1973; Blake et al., 1981) is stronger under dimmer conditions, suggesting that summative processes, rather than stereopsis, could be responsible for the binocular advantage observed by CuQlock-Knopp and colleagues under no-moon conditions.

Given the inconsistent findings in the literature, and the improvements in tube quality in recent years, a renewed assessment of depth perception through NVGs was felt warranted.

## The present study

The approach taken in the present study builds upon past work examining depth perception through NVGs. In Experiment 1, a Howard-Dolman apparatus was used to assess stereoacuity with unaided vision under day conditions, as well as through NVGs under low-light conditions. In both cases, performance was assessed monocularly and binocularly.

In addition to the more clinical assessment of stereoacuity using the Howard-Dolman apparatus that isolates the contribution from binocular sources, a more operationally relevant reach-and-grab task was performed in Experiment 2 in which participants reached for and removed objects from a table. Performance was assessed under well-lit conditions with unaided vision, as well as under low-light conditions through NVGs. As in the Howard-Dolman apparatus task, performance was assessed monocularly and binocularly. This more operationally relevant task sought to determine whether binocular depth cues enhance performance under more ecologically valid (Gibson, 1979) conditions in which both monocular and binocular depth cues are available.

Extending past work, in the present study the focal distance of the NVG was also manipulated. In previous studies using the Howard-Dolman apparatus, the NVG was always adjusted so that the fixed rod was in sharp focus. While these are perhaps the most likely conditions under which evidence for stereopsis is to be found, it does not reflect how much of the visual scene is experienced through NVGs. When employed outdoors—for example, by dismounted soldiers on a patrol—the NVG is typically focused to infinity. As the soldier moves through space, more-distant scene elements are in relatively sharp focus, contributing to situational awareness and allowing potential threats to be detected from farther away. However, a direct consequence of this choice of focal distance is that nearer scene elements, those within roughly 15 m, are out of focus, becoming increasingly more so as the distance decreases (Verona & Rash, 1989).

This is important because stereopsis is most potent at relatively close distances (< 20 m; Cutting & Vishton, 1995), contributing significantly to effective and efficient mobility (Hayhoe et al., 2009), which depends on an accurate spatial representation of one’s immediate vicinity. So, if the choice of focal distance results in significant defocusing of scene elements in one’s immediate vicinity (i.e., where stereopsis is most effective), and if defocusing disproportionately affects stereoacuity (Goodwin & Romano, 1985; Westheimer & McKee, 1980), the consequences for depth perception may be significant. In fact, consistent with this line of reasoning, Knight et al. (1998) attributed the residual loss in depth perception through NVGs that they observed to reduced visual acuity. Therefore, one would expect further reductions in stereoacuity for scene elements off the focal plane.

To assess this possibility, in the present study testing was conducted at three levels of focus: infinity, near (7–8 m) and ideal (optimized to the task). As previously mentioned, the infinity condition was included to reflect how NVGs are typically focused by dismounted soldiers in an outdoor context. When working indoors, a nearer focus is typically employed. Based on recent field work in which a room-clearing task was performed (Tombu et al., 2024), a 7-m or 8-m focus distance was chosen as the near focus condition to reflect how NVGs are configured in an interior context. Finally, an ideal condition was chosen to replicate past work and to have an upper bound on stereopsis under NVGs.

Recognizing the issues associated with the manual focus of NVGs, some soldiers choose to employ a different focus distance for each eye (typically one near and one far). In so doing, they ensure that most of the world is in relatively good focus for one eye. While this approach makes sense in terms of image clarity, it may have negative consequences for stereoacuity. Goodwin and Romano (1985; see also Levy & Glick, 1974) found that optically defocusing one eye while leaving the other unimpaired significantly degraded stereopsis—in fact, more so than defocusing both eyes by the same amount. To directly assess this configuration, a mixed focus binocular condition was included in Experiment 1 to examine its effect on stereoacuity through NVGs.

## Experiment 1: Assessing stereopsis through NVGs

The primary goal of Experiment 1 was to assess stereopsis through NVGs under different focal configurations. Testing was conducted using a Howard-Dolman apparatus at 3 m.^1^

A total of nine conditions were assessed (see Table 1, in the Design section, below). These included unaided binocular and monocular conditions under bright conditions to establish an upper and lower bound on performance, as well as seven NVG conditions. Depth discrimination thresholds through NVGs were collected at three focal distances: infinity, 7 m,^2^ and 3 m. Each of these conditions was assessed monocularly and binocularly. Finally, a mixed focal distance binocular condition was included in which one tube was focused to infinity, while the other was set to 3 m.

**Table 1:** Experimental design, Experiment 1

		Eye condition factor	
	Factor levels	Monocular	Binocular
Viewing condition factor	Unaided	Unaided Mono	Unaided Bino
	NVG Ideal	NVG Ideal Mono	NVG Ideal Bino
	NVG 7 m	NVG 7 m Mono	NVG 7 m Bino
	NVG Infinity	NVG Inf Mono	NVG Inf Bino
	NVG Mixed		NVG Mixed

In addition to collecting depth discrimination thresholds, visual acuity was also measured. Unaided visual acuity was evaluated both monocularly and binocularly using EDTRS charts from 4 m under the same bright lighting conditions as employed for the Howard-Dolman apparatus task.

Two approaches were taken to evaluate visual acuity through NVGs. The first employed a Hoffman Engineering (Stamford, CT, USA) ANV-20/20 Infinity Focus System and allowed visual acuity to be assessed at two ‘standard’ lighting levels: starlight and ¼-moon. Performance was assessed for each monocularly and binocularly. The ANV-20/20 simultaneously presents participants with nine sets of test gratings (each set contains a horizontally striped and a vertically striped grating) at progressively finer resolution (20/70, 20/60, 20/50, 20/45, 20/40, 20/35, 20/30, 20/25, 20/20). After adjusting the NVG to bring the gratings into sharp focus, the participant indicates the smallest grating for which they can still make out the stripes. Although this method provides a fast and effective way of measuring performance under standardized lighting conditions, the assessment is relatively coarse (measuring acuity in 5-point increments on the Snellen denominator) and subjective in nature and cannot easily be used to measure visual acuity at different levels of illumination.

In order to more precisely assess visual acuity under the lighting employed during the Howard-Dolman apparatus task, visual acuity through the NVG was also assessed under these lighting conditions using the EDTRS charts. Paralleling the unaided vision testing, visual acuity was assessed monocularly and binocularly from 4 m. To quantify the impact of the defocusing manipulation on visual acuity, performance was also evaluated at each level of focal distance (infinity, 7 m, 3 m) from 3 m (the same distance from which the Howard-Dolman apparatus task was performed) binocularly. The EDTRS charts provide an objective, fine-grained assessment of visual acuity under the same lighting conditions for which the Howard-Dolman apparatus task was conducted and therefore accurately quantify the effective visual acuity achieved at each level of focal distance employed for the Howard-Dolman task.

### Methods

#### Participants

Twenty infantry-qualified Canadian Armed Forces (CAF) members from 4 Division took part in Experiment 1. Eight were Regular Force members, while the remaining 12 were members of the Reserve Force. Nineteen participants were men and one was a woman. Three were senior noncommissioned members (NCMs; Sergeants or higher) and 17 were junior NCMs. On average, participants had 5 years 2 months of time in the military (standard error [*SE*] = 1 year 3 months). Four participants had completed at least one overseas deployment. The average participant age was 27 years 10 months (*SE* = 1 year 9 months). All participants were run during the winter of 2024 (February–March).

All participants would have received both classroom and field NVG training as part of their infantry qualification courses. Additional NVG experience would have been acquired on exercises over the course of their career, with opportunities being more frequent for Regular Force members. It is worth noting that NVG experience would likely be limited to the monocular and biocular systems (PVS-14 and PVS-7 respectively) currently in service with the Canadian Army.

Seventeen participants were right-eye dominant and three were left. Three participants wore glasses or contacts to correct visual acuity to normal. The average interpupillary distance, as measured with a Rheichert (Buffalo, NY, USA) digital pupilometer, was 63.1 mm (*SE* = 0.616 mm). Visual acuity was confirmed monocularly (dominant eye) and binocularly (with vision correction if required) using EDTRS charts from 4 m under well-lit (typical office lighting) conditions. Mean binocular acuity was − 0.15 (logMAR, *SE* = 0.025; Snellen equivalent 20/14.16) and mean monocular acuity was − 0.075 (logMAR, *SE* = 0.031; Snellen equivalent = 20/16.83). More information on visual acuity is presented in the Results section, below.

#### Testing room and equipment

All testing was conducted in a windowless *L*-shaped basement storage room. All walls, the floor and the ceiling were white/off-white. The room was 7.06 m long and 5.87 m wide on the wide end of the room, and 4.03 m wide at the narrow end. The room was narrow for 3.43 m and wide for 3.63 m. Double doors were located along the wall opposite the long (7.06 m) wall in the wide section of the room. The gaps around one of the doors were taped off to block light from the adjacent hallway. A 1.54-m-high room partition was set up to cut off the area around the doorway from the rest of the room, leaving a roughly 7.06 × 4.03 m area where the testing was conducted. Of this space, a 7.06 × 2.93 m area was cleared and used to conduct the experiment (two desks, boxes and assorted equipment occupied some of the remaining space, all of which was well out of the line of sight used for the experiment). The partition blocked most light seeping in through the gaps in the non-taped-off door. The ceiling height throughout the room was 2.13 m.

The room was lit by fluorescent lights built into the drop ceiling. These provided ambient lighting conditions typical for office spaces. When testing under NVGs, the overhead lights were turned off and the only light was provided by the near infrared LED illuminator from a Wilcox (Newington, NH, USA) Raid-X laser aiming device pointed at the (vinyl tile) floor mounted on a tripod by the door in the partitioned-off portion of the room. Because the Raid-X was located on the other side of the partition and pointed at the floor, the light arriving at the Howard-Dolman apparatus was indirect and diffuse having to reflect off the floor, walls and ceiling before arriving at the Howard-Dolman apparatus. The Raid-X was operated by the experimenter using an extended (roughly 1.83-m-long) remote switch that allowed the experimenter to operate the illuminator without having to disturb the partition. The ambient light level at the Howard-Dolman apparatus, as measured by a Hoffman Engineering ANV-410, was 1.75 mlux, roughly equivalent to a moonless clear night sky with airglow.

A Bernell Corporation (Mishawaka, IN, USA) Howard-Dolman apparatus was positioned on an adjustable-height table so that it could be viewed along the long axis of the room. The fixed post of the Howard-Dolman apparatus was positioned 2.11 m from the back wall, and 0.68 m from the side wall. The bottom of the viewport was 1.09 m from the floor. The Howard-Dolman apparatus measured 0.47-m long × 0.23-m wide. A 15.24-cm × 5.08-cm viewport was located along one of the short ends of the Howard-Dolman apparatus, allowing an observer to see the fixed and adjustable posts from the observation point located 3 m (from the fixed post) away. The Howard-Dolman apparatus was open on top to allow reflected light from the laser aiming device or from the overhead lights to illuminate the posts. In neither case, were shadows visible when the posts were observed from the observation point. The Howard-Dolman apparatus was constructed from black ABS plastic, with white posts. Each post was 7.7 mm in diameter, subtending 529 arcsec at 3 m. When viewed from the observation point, the posts were only visible through the viewport, with the black back wall of the Howard-Dolman apparatus providing a high contrast background. The separation between the posts was 10 cm. White strings connected to the adjustable post extended to the observation point. These strings were not visible to the participant within the Howard-Dolman apparatus, connecting to the adjustable post at its base, which was out of the line of sight of the participant. The participant could move the adjustable post backward or forward by pulling on the strings. The maximum travel of the adjustable post was 197 mm behind and 200 mm in front of the fixed post. Separation between the posts in depth was indicated on the base of the inside of the Howard-Dolman apparatus for 50 mm in front of and behind the fixed post. A ruler was used to measure additional distance beyond this range.

Participants performed the task seated at a table equipped with a University of Houston College of Optometry Tech (Houston, TX, USA) chin rest. The chin rest was positioned so that the participant’s chin was 1.05 m from the floor and centered left to right on the middle of the viewport. With this set up, the participant’s eyes were slightly higher than the top of the viewport (~ 3 cm), but neither the base nor the top of either post was visible from that vantage point. At this height the participant was able to sit comfortably while performing the task.

Two Precision Vision (Woodstock, IL, USA) plastic EDTRS 4 m high-contrast visual acuity charts (Charts 1 and 2) were mounted on the short wall of the room behind and to the left (as viewed from the observation point) of the Howard-Dolman apparatus, with the top row of the chart 1.78 m from the floor. The floor was taped to establish 3-m and 4-m viewing lines from the charts. The 3-m line was used for NVG visual acuity testing and for focusing the NVG to 3 m. The 4-m line was used for NVG and unaided visual acuity testing. The charts were also used to assist with focusing the NVG to 7 m. To do so, the participant stood with the back of their head against the wall behind the observation point and adjusted the focus of each tube assembly to bring the charts into sharp focus. All visual acuity testing and focusing was performed from standing.

A Hoffman Engineering ANV-20/20 was set up on a table behind and to the left of the observation point. The ANV-20/20 was used to set the NVG focus to infinity, and to assess NVG visual acuity under starlight and ¼-moon conditions.

Each participant wore a properly fitted Team Wendy (Cleveland, OH, USA) Exfil Carbon bump helmet (Rail 2.0) with a built-in shroud. Depending on the size, the Exfil Carbon weighs between 750 and 760 g. The NVG was mounted to the helmet using a Wilcox push-to-overcome G24 mount. The G24 weighs 161 g. The binocular NVG used for all testing was a Thales Bonie lightweight (LW). Each of the Bonie’s tube assemblies can be focused from 20 cm to infinity, allows for diopter adjustment in a range from − 6 to + 3 D, and can be independently stowed, allowing the Bonie to also be used in a monocular configuration. The Bonie was equipped with high-performance third-generation Harder Digital (Woltersdorf, Germany) white phosphor tubes, which could be adjusted to suit the interpupillary distance and preferred eye relief of the user. When employed in a monocular configuration, some participants opted to use a lens cover over the tube on their nondominant eye, while others stowed it. This choice was largely driven by interference between the stowed tube and the helmet for those who wore the NVG very close to their face. In neither case could anything be seen with the non-dominant eye when monocularly configured. The Bonie weighs 540 g. A 320 g counterweight was Velcroed to the back of the helmet to offset the weight of the NVG, providing a more comfortable center of balance for the weight borne by the participant’s head and neck.

A black plastic eye patch was worn over the non-dominant eye of the participant when conducting unaided monocular testing.

#### Design

The experiment was set up as an incompletely crossed two-factor within-subjects design. The first factor was viewing condition, with five levels: Unaided, NVG Ideal, NVG 7 m, NVG Infinity, and NVG Mixed. The second factor was eye condition, with two levels: Monocular (dominant eye), and Binocular. All levels of the viewing condition factor except for NVG Mixed were fully crossed with both eye condition levels. The NVG Mixed level of the viewing condition factor was only performed binocularly. See Table 1 for a summary of the factors and levels examined. Each participant completed the task in each of the nine experimental conditions, with condition order counterbalanced across participants using a Williams Design Latin Square on the viewing condition factor.

With five levels of the viewing condition factor, a complete counterbalance is achieved every 10 participants. Two groups of 10 participants each, completed the experiment, for a total of 20 participants. One participant from each group was assigned to each condition order (i.e., each group consisted of a complete counterbalance). The monocular and binocular runs at each level of the viewing condition factor were always performed sequentially, one after the other. One group of participants always performed the monocular run first, followed by the binocular run, while the other group did the binocular run first, followed by the monocular run. As such, the average serial order position for each condition was five.

#### Procedure

Participants completed the experiment individually over two sessions. The first session was scheduled for 2 h, with 1.5 h allotted to the second session. Most participants completed their sessions more quickly, however (typical total time ~ 3 h).

Upon arriving for their first session, participants were briefed on the purpose of the study and informed consent was obtained. They were then briefed on how the Howard-Dolman apparatus worked and provided an overview of the tasks to be completed as part of the experiment. Unaided (i.e., natural vision, with vision correction if required) visual acuity was then assessed monocularly and binocularly using the EDTRS charts from 4 m under well-lit conditions (with the overhead lighting on). Half the participants were tested on Chart 1 binocularly and Chart 2 monocularly, with the mapping reversed for the other half of participants. Participants always began on the top row of the chart, reading off the letters from left to right, and worked their way down the chart until they could no longer make out any letters. The experimenter encouraged the participants to guess if they thought they could make out a letter but were unsure. LogMAR scores (line values indicated on the chart for the lowest complete line read, − 0.02 per additional letter) were recorded by the experimenter.

The participants were then provided an overview of the helmet, NVG mount and NVG to be used in the experiment. They were instructed on how to properly adjust each component to comfortably align both tube assemblies with their eyes (fore–aft, up–down and tilt adjustments on the NVG mount, left–right adjustment on each tube assembly of the NVG). They were then provided with an overview of the operation of the NVG, including on/off, diopter adjustment, focus adjustment, and stowing of the tube assemblies. With the lights out and the near infrared illuminator on, the NVG was turned on and first adjusted for diopter, followed by focus for each tube assembly from the 4-m line using the EDTRS charts to assist. Considerable time was taken to achieve a well-aligned, sharp image before moving on to the next part of the experiment. Although the gain could be adjusted, participants were instructed to leave it at the default level throughout testing.

With the NVG properly fitted and a clear image achieved, participants moved to the ANV-20/20 for visual acuity testing through the NVG. Participants first adjusted the focus of each tube assembly to infinity by bringing the test grating of the ANV-20/20 into sharp focus. Binocularly and then monocularly (dominant eye), they indicated the highest frequency grating for which they could just make out the lines of the test grating. Results were recorded by the experimenter.

Next, the participant returned to the 4 m line and again adjusted the focus of each tube assembly to bring the EDTRS charts into sharp focus. Visual acuity through the NVG was again assessed binocularly and monocularly, this time with the EDTRS charts (Charts 1 and 2). As with the unaided visual acuity testing, half of the participants were assessed binocularly on Chart 1 and monocularly on Chart 2, with the mapping reversed for the other half of the participants. The same procedure as with unaided visual acuity testing was followed. Although the same charts were used for unaided and NVG visual acuity testing, the roughly 5–10-min gap and intervening tasks (NVG setup and ANV-20/20 testing) seemed to be sufficient to limit memory effects on the second round of visual acuity testing using the EDTRS charts.

The NVG was then turned off and stowed, and the lights were turned back on so that participants could familiarize themselves with the operation of the Howard-Dolman apparatus. Participants conducted two unaided binocular trials and two unaided monocular trials under typical office lightning, both with feedback, to become acquainted with the task and the operation of the Howard-Dolman apparatus. They were also instructed to rely on visual cues to perform the task, and not to game it by moving the adjustable post all the way back, then all the way forward and attempting to split the difference (or any other nonvisual strategy). Although participants did run the adjustable post into either the front stop or the back stop on occasion during testing, especially in monocular conditions, these appeared to the experimenter, who was present throughout, to be honest errors caused by task difficulty and not attempts to game the task. Two binocular and two monocular practice trials through the NVG were then conducted with the NVG focused to 3 m. All focus adjustments were always performed from the 3-m/7-m lines using the EDTRS charts to achieve a sharp image in ideal and 7-m focus conditions, respectively, and using the ANV-20/20 in the infinity focus conditions. In the NVG Mixed condition, the dominant eye was focused to infinity, while the non-dominant eye was focused to 3-m. Additional practice trials were permitted if necessary.

With setup, training, and visual acuity testing complete, experimental blocks were next completed in accordance with the counterbalanced run order. To begin a block, participants first set up the NVG according to the condition assignment. For Unaided conditions, the helmet and NVG were still worn, with the NVG stowed, to keep the weight across conditions consistent. In the Monocular Unaided condition, the participant also wore an eye patch over their nondominant eye. The experimenter confirmed that the eye patch occluded vision from the nondominant eye before testing began.

Each block consisted of 10 alignment trials. Each trial began with the participant releasing the strings used to adjust the adjustable post of the Howard-Dolman apparatus, the experimenter blocking the viewport^3^ with a piece of cardboard and adding slack to the strings by pulling them toward the Howard-Dolman apparatus so that the participant could not use the position of the strings to assist with the next trial. With the viewport still blocked, the experimenter randomly positioned the adjustable post roughly up to 70 mm in front of or behind the fixed post. With the trial ready to begin, the participant assumed their position on the chin rest, the experimenter removed the cardboard blocking the viewport and retreated to a seat roughly 3 m from the Howard-Dolman apparatus perpendicular to the line of observation. At this distance the experimenter was well out of the line of sight of the participant, and in fact outside of the field of view of the participant when using the NVG. The participant then adjusted the position of the adjustable post until they believed it was aligned with the fixed post, released the strings and sat back from the chin rest. At this signal, the experimenter returned to the Howard-Dolman apparatus, blocked the viewport with the cardboard and recorded the offset between the fixed and the adjustable posts (in mm). No feedback was provided. In NVG conditions, the experimenter was equipped with a monocular NVG so that they could monitor the participant. For offset measurement, the experimenter stowed the NVG and used a green light from a headlamp to make and record the measurement. This procedure was repeated until all 10 trials in a block had been completed.

Participants were given an opportunity to take a break between blocks, which was taken as needed (typically an additional couple of minutes to rest their eyes or to adjust/remove their helmet to address neck strain). Most participants (13) completed five blocks in Session 1, with the number of blocks completed ranging from three to six. The remaining blocks were completed in Session 2. Sessions 1 and 2 were always completed on different days of the same week.

Session 2 began with properly fitting and adjusting the helmet, mount and NVG to the participant using the same procedure used in Session 1. With a sharp image achieved, testing resumed where it left off. Once all nine experimental blocks had been completed, a final series of NVG visual acuity testing was performed to determine the impact of the change in focus distance on visual acuity for elements at the testing distance of 3 m. For this test, participants first adjusted the focus of both tube assemblies to infinity using the ANV-20/20 and then moved to the 3-m line and performed a visual acuity test using EDTRS Chart 1. They then moved to the 7-m line and brought the chart into sharp focus before returning to the 3-m line and conducting a second acuity test, again using EDTRS Chart 1. Finally, the participants adjusted the focus of the NVG to bring the chart into sharp focus from the 3-m line and performed a third acuity test using EDTRS Chart 1. Because participants always progressed from the worst focus distance (for visual acuity at 3 m) to the best, and because the impact on visual acuity moving from focus distance to focus distance was so large, there was little chance that previous exposure to the same chart affected visual acuity performance. The EDTRS charts are designed to be used from 4 m, while this block of testing was conducted from 3 m to match the test distance used in the Howard-Dolman apparatus task. To correct for the change in distance, 0.125 was added to each logMAR score calculated for 4 m (line value of the lowest line completed, − 0.02 per additional letter).

#### Analysis

##### Visual acuity from 4 m with EDTRS charts

Unaided and NVG visual acuity scores were analyzed separately. In each case monocular and binocular logMAR scores for each participant were compared using a pairwise *t* test. Means in each condition were calculated by taking the average of the logMAR scores. LogMAR scores were then converted to Snellen scores for ease of interpretation.

##### Visual acuity with ANV-20/20

Snellen scores in each condition were first converted to logMAR scores for each participant. Monocular and binocular scores at each light level (starlight, ¼-moon) were then compared using a pairwise *t* test. Means in each condition were calculated by taking the average of the logMAR scores. LogMAR scores were then converted to Snellen scores for ease of interpretation.

##### Visual acuity from 3 m with EDTRS charts

A one-way analysis of variance (ANOVA), with focus distance (ideal [3 m], 7 m, infinity) as the factor, was performed on logMAR visual acuity scores. Means in each condition were calculated by taking the average of the logMAR scores, which were then converted to Snellen scores for ease of interpretation.

##### Depth discrimination thresholds

For each participant in each condition (viewing condition × eye; see Table 1) a depth threshold in mm was calculated by taking the mean of the absolute value of the separation between the adjustable and fixed post (in mm) on the ten alignment trials. A depth threshold in arcsec was then calculated using the following formula:

$${Threshold}_{arcsec}=IPD\times {Threshold}_{mm}\times 206265/{d}^{2},$$

where IPD is the interpupillary distance of the participant, Threshold_mm_ is the threshold in mm and d is the viewing distance to the fixed post (3 m), all in metres.

Thresholds (in arcsec) were then, submitted to planned tests to address a priori questions of interest (Table 2).

**Table 2:** Planned comparisons, Experiment 1

Question	Comparison
What is the extent of baseline stereopsis?	Pairwise *t* test Unaided (Mono vs. Bino)
Do NVGs provide stereopsis, even when defocused?	3 × pairwise *t* test: NVG Ideal (Mono vs. Bino) NVG 7 m (Mono vs. Bino) NVG Infinity (Mono vs. Bino)
Is NVG stereopsis as good as natural stereopsis?	3 × pairwise *t* test: Unaided Bino versus NVG Ideal Bino Unaided Bino versus NVG 7 m Bino Unaided Bino versus NVG Infinity Bino
What is the effect of defocusing on stereoacuity?	3 × pairwise *t* test: NVG Ideal Bino versus NVG 7 m Bino NVG ideal Bino versus NVG Infinity Bino NVG 7 m Bino versus NVG Infinity Bino
Does an NVG with mixed focus provide stereopsis?	Pairwise *t* test Unaided Mono versus NVG Mixed

With nine conditions, the experimental design provides 8 degrees of freedom and therefore allows up to eight tests before a correction for multiple comparisons is required. Because 11 planned comparisons were undertaken, the threshold for significance was adjusted from 0.05 to 0.03636 (0.05/(11/8)).

With 20 participants, two-tailed matched-sample *t* tests can detect a medium effect size difference (Cohen’s *d* = 0.5) with 0.56 power, and a large effect-size difference (*d* = 0.8) with 0.92 power.

A Greenhouse–Geisser correction for violations of sphericity that adjusts the degrees of freedom was applied to all ANOVAs. Raw data can be obtained by contacting the author. This experiment was not preregistered.

### Results

#### Visual acuity at 4 m with EDTRS charts

**Fig. 1:**
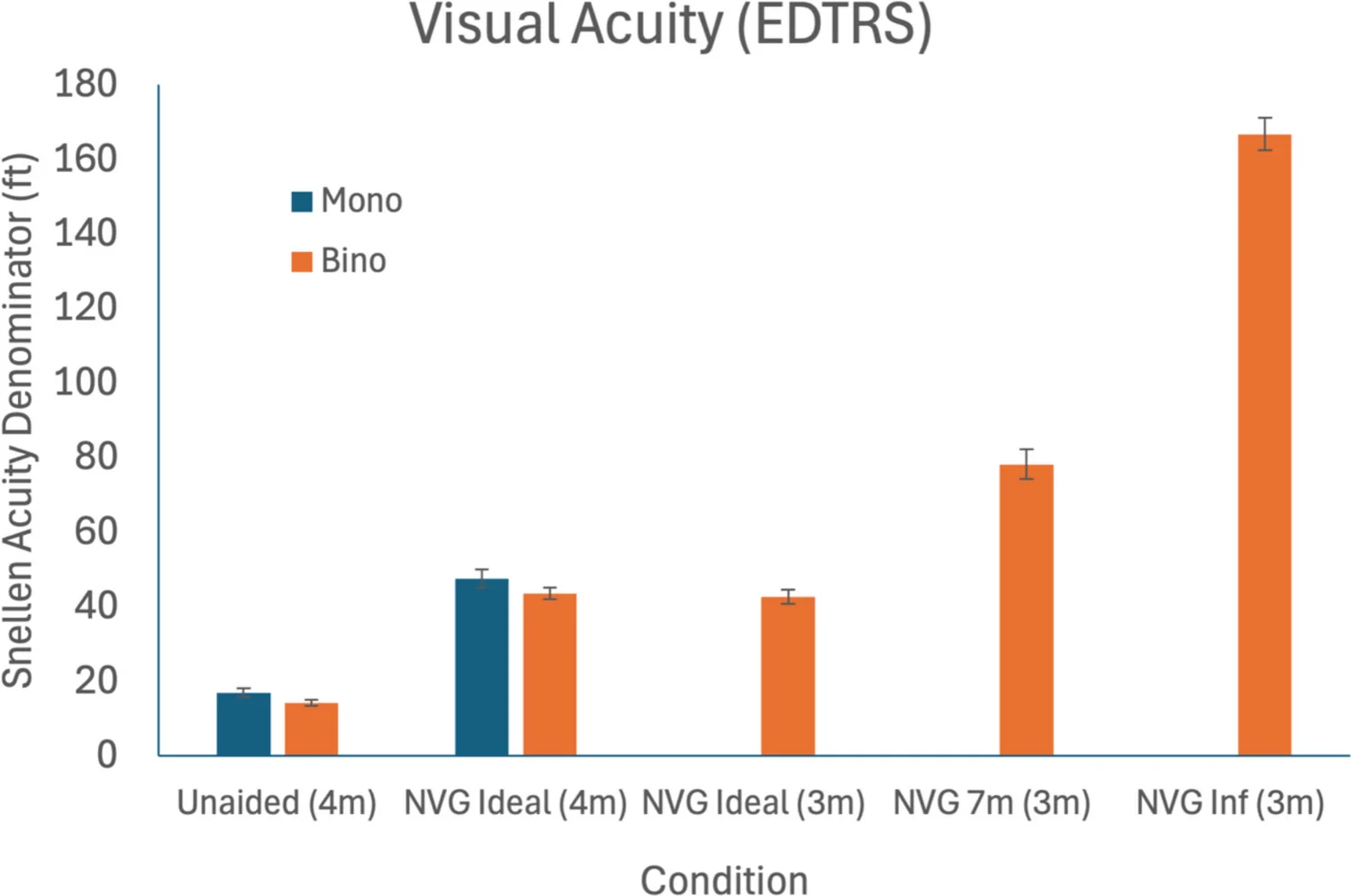
Visual acuity as assessed with EDTRS charts. Visual acuity was assessed using standard EDTRS charts in the Unaided and NVG Ideal (ideally focused) conditions from 4-m monocularly and binocularly (four left bars). Visual acuity was also assessed from 3-m binocularly in the NVG Ideal (focused to 3 m), NVG 7 m (focused to 7 m), and NVG Infinity (focused to infinity) conditions (three right bars). Individual scores were measured in logMAR and averaged before converting to Snellen Acuity. A 0.125 adjustment was added to the logMAR acuity scores when assessed from 3 m to correct for the fact that testing was conducted at a reduced distance. A small but significant binocular advantage was observed in both the Unaided (4 m) and NVG Ideal (4 m) conditions. Acuity scores derived from assessments at 4 m (with no correction) and at 3 m (with a 0.125 correction) produced almost identical results (NVG Ideal [binocular] at 4 m and 3 m). Visual acuity degraded significantly as the focus distance departed from the observation distance. All NVG testing was conducted at 1.75 mlux. Unaided testing was performed under well-lit conditions. Error bars show the standard error of the mean

Visual acuity results, as assessed with the EDTRS charts from 4 m are presented in the left portion of Fig. 1.

Although visual acuity in the unaided condition under well-lit conditions was excellent both monocularly (20/16.83) and binocularly (20/14.16), a *t* test revealed a significant binocular advantage, *t*(19) = 4.40, *p* < 0.001.

Similarly, testing conducted through NVGs under low-light conditions (1.75 mlux) also revealed a binocular advantage, *t*(19) = 2.10, *p* < 0.05. Visual acuity improved from 20/47.43 in the monocular condition to 20/43.55 in the binocular condition.

Both results are consistent with participants benefiting from binocular summation, suggesting that summative processes resulted in improved image processing in the visual system.

#### NVG visual acuity with ANV-20/20

NVG visual acuity was also assessed at two standard illumination levels (starlight and ¼-moon) monocularly and binocularly using the ANV-20/20. Under starlight conditions, a significant binocular advantage was observed, t(19) = 2.14, p < 0.05, with acuity improving form 20/39.76 monocularly to 20/37.75 binocularly.

Although a similar pattern of results was observed at ¼-moon illumination (monocular visual acuity = 20/29.56, binocular visual acuity = 20/27.66), the difference failed to reach the threshold for significance, *t*(19) = 1.84, *p* < 0.09.

#### NVG visual acuity at 3 m with EDTRS charts

NVG visual acuity, as assessed with the EDTRS charts from 3 m under low-light conditions (1.75 mlux) are presented in the right portion of Fig. 1.

Performance was assessed binocularly at three focal distances: ideal (3 m), 7 m, and infinity. The results of the one-way ANOVA indicated a significant impact of defocusing the NVG, *F*(1.92, 36.40) = 387.39, η_p_^2^ = 0.953, *p* < 0.001. At ideal focus, visual acuity was 20/42.56, dropping to 20/78.17 at 7 m, and 20/166.74 at infinity focus.

These results confirm that the manipulation of focal distance had a strong and significant effect on visual acuity performance, paving the way for the assessment of the impact of defocusing the NVG on stereoacuity.

#### Depth discrimination thresholds

**Fig. 2:**
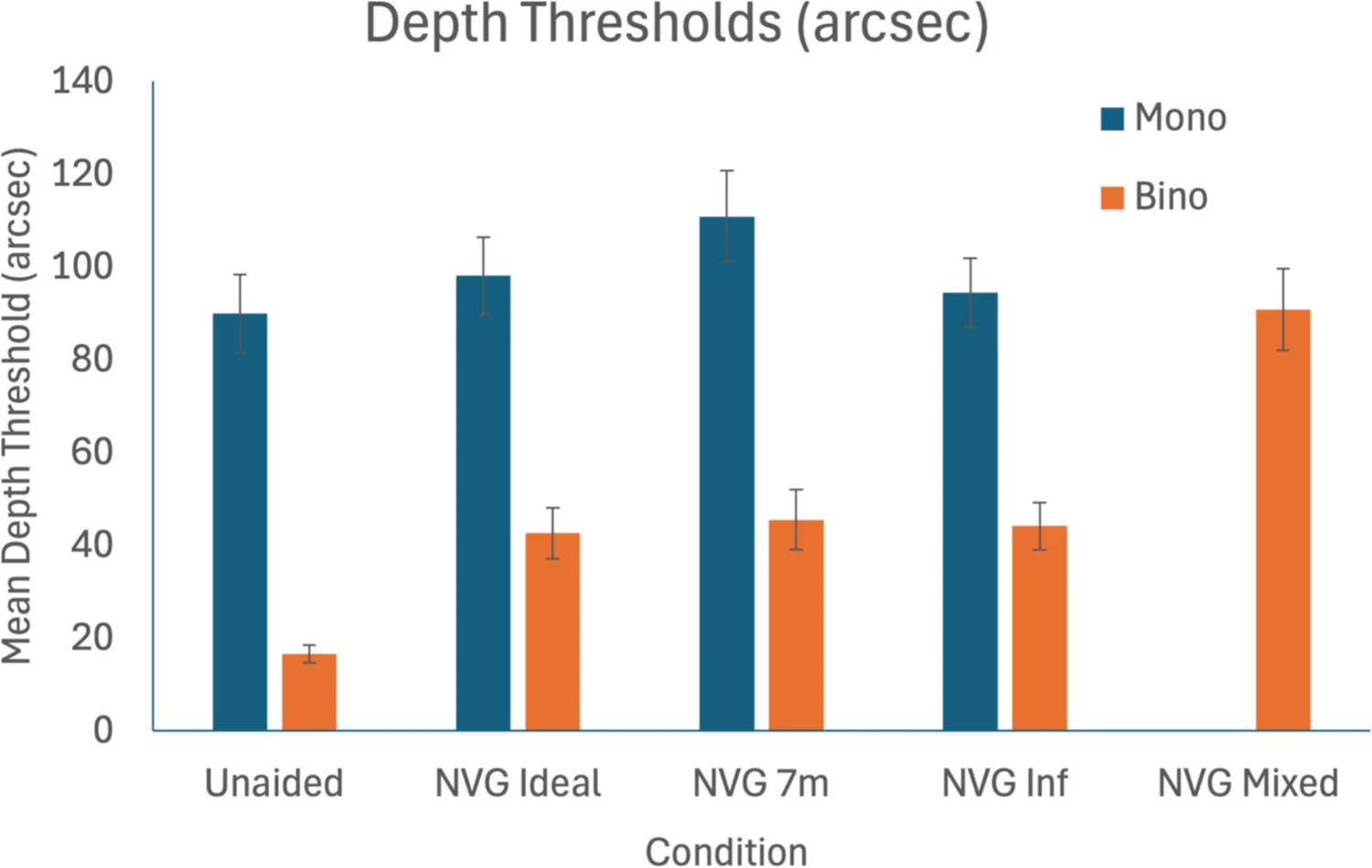
Depth thresholds as a function of viewing condition. Depth thresholds were assessed using a Howard-Dolman apparatus from 3 m. NVG testing was conducted at 1.75 mlux and Unaided testing was performed under well-lit conditions. Performance was assessed monocularly and binocularly in the Unaided, NVG Ideal (NVG focused to 3 m), NVG 7 m (NVG focused to 7 m), and NVG Inf (NVG focused to infinity) conditions, and binocularly in the NVG Mixed condition (one tube focused to infinity, one tube focused to 3 m). A strong binocular advantage was observed in all conditions that were assessed monocularly and binocularly. Thresholds were significantly lower in the Unaided binocular condition than in the other binocular conditions. Change in focus had no impact on binocular thresholds in the NVG conditions so long as isoacuity was maintained. When isoacuity was not maintained, binocular depth thresholds degraded to monocular levels. Error bars show the standard error of the mean

Depth discrimination thresholds in arcsec for each condition are presented in Table 3 and shown in Fig. 2.

**Table 3:** Depth discrimination thresholds by condition (arcsec)

Viewing condition	Monocular (*SE*)	Binocular (*SE*)
Unaided	89.96 (8.71)	16.64 (1.99)
NVG Ideal (3 m)	98.09 (38.13)	42.62 (5.67)
NVG 7 m	110.85 (10.12)	45.53 (6.67)
NVG Infinity	94.49 (7.61)	44.14 (5.15)
NVG Mixed		90.80 (9.05)

##### What is the extent of baseline stereopsis?

Baseline stereopsis was assessed by comparing Unaided depth discrimination thresholds in the monocular and binocular conditions using a pairwise *t* test. As expected, a significant binocular advantage was detected, *t*(19) = 8.13, *p* < 0.001, with thresholds decreasing from 89.96 arcsec in the monocular condition to 16.64 arcsec in the binocular condition.

##### Do NVGs provide stereopsis, even when defocused?

Parallel pairwise *t* tests were conducted in the NVG Ideal, NVG 7 m, and NVG Infinity viewing conditions. In all cases, a significant binocular advantage was detected, all *t*(19) values > 7.35, *p* values < 0.001. Binocular performance ranged from 42.62 to 45.53 arcsec, while monocular performance ranged from 94.49 to 110.85 arcsec.

##### Is NVG stereopsis as good as natural stereopsis?

To assess whether the level of stereopsis provided at each NVG focus distance reached that provided under Unaided conditions, each of the binocular NVG conditions (Ideal, 7 m, Infinity) were compared against Unaided Binocular performance using a pairwise *t* test. In all cases, stereoacuity was significantly better Unaided than through the NVG, all t(19) values > 4.23, *p* values < 0.001.

##### What is the effect of defocusing on stereoacuity?

To see if stereoacuity improved as the image became more sharply focused, pairwise *t* tests were conducting comparing each of the binocular NVG conditions (NVG Ideal, NVG 7 m and NVG Infinity). Results revealed no improvement with improving focus, all *t* values < 1, *p* > 0.63.

##### Does an NVG with mixed focus provide stereopsis?

Finally, stereoacuity in the NVG Mixed viewing condition, where the dominant eye was focused to infinity and the nondominant eye was focused to 3 m, was compared against the Unaided Monocular condition using a pairwise *t* test. Results indicated no improvement relative to the monocular baseline,^4^*t*(19) = − 0.07, *p* > 0.94.

### Discussion

As is typically observed, significant depth discrimination benefits were found when participants performed the Howard-Dolman task binocularly compared with monocularly under well-lit, natural viewing conditions.

Although a binocular advantage was also observed through NVGs, performance did not approach the level achievable under natural viewing conditions. Providing binocular inputs improved thresholds by upwards of 56% relative to monocular performance but remained over 2.5 times worse than the level attained in the Unaided Binocular condition. These results are in good agreement with those of Knight et al. (1998), despite being conducted with more modern NVGs and under darker conditions.

Interestingly, changes in the depth of focus had little influence on stereopsis, so long as both eyes were equally affected (isoacuity). Even when the image quality was seriously degraded, as low as 20/166, the degree of stereoacuity provided by binocular NVGs held steady. This result deviates from the literature examining the effects of visual acuity on stereoacuity under photopic conditions (Donzis et al., 1983; Goodwin & Romano, 1985; Westheimer & McKee, 1980). In those studies, significant degradation of stereoacuity accompanied decreases in visual acuity, with stereopsis being virtually eliminated at levels of visual acuity worse than 20/100. In contrast, herein stereoacuity was essentially unaffected by changes in visual acuity in a range from 20/43 to 20/166. Significant differences in test methodology (e.g., contour stereo tests versus real depth), conditions of test (e.g., natural viewing versus through NVGs) and how visual acuity was manipulated (e.g., cycloplegic intervention and refractive lenses versus depth of focus) were however present, which may explain the divergent results.

One past explanation for the impairment in stereoacuity through NVGs (even when properly focused) relative to natural viewing is that the visual acuity provided by NVGs is not sufficient for proper stereoacuity, resulting in reduced or eliminated stereopsis (Knight et al., 1998). While this explanation is consistent with the literature relating visual acuity to stereoacuity under natural viewing conditions, it does not fit well with the observed results of this study. Although a reduction in stereoacuity accompanied a reduction in visual acuity moving from binocular unaided to binocular NVG with ideal focus viewing, no further decrease in stereoacuity was observed as the focal distance was manipulated and visual acuity worsened. This result suggests that other factors (e.g., reduced contrast, reduced luminance) may be responsible for the initial reduction in stereoacuity moving to NVG viewing. Further stereoacuity testing under brighter conditions where visual acuity through NVGs approaches normal (20/20) may provide insight into the relationship between visual acuity and stereoacuity through NVGs.

From an operational perspective, the preservation of stereopsis for scene elements away from the focal plane of the NVG is a welcome result. Despite poor visual acuity in these zones, binocular cues continue to provide information to the visual system that can be used to ascertain depth and therefore facilitate performance for tasks that rely upon depth perception.

Although stereopsis was partially preserved when the focal distance of the NVG tubes was matched for each eye (isoacuity), this was not the case when mixed focal distances were employed. This result is consistent with past work, which has found that monocular visual acuity degradation has a larger effect on stereoacuity than binocular degradation (Donzis et al., 1983; Goodwin & Romano, 1985; Levy & Glick, 1974; Westheimer & McKee, 1980).

Even though focusing each eye to a different depth means that most scene elements will be in focus for at least one eye, operators should be aware that doing so has significant negative repercussions on stereoacuity (and eye fatigue) and may effectively eliminate the depth perception advantage provided by binocular NVGs when focused to the same distance.

On the whole, the results of Experiment 1 demonstrate that binocular NVGs provide improved depth discrimination relative to monocular configurations, albeit at levels significantly reduced relative to natural viewing. Furthermore, improved stereoacuity extends to scene elements off the focal plane, an important observation given that unlike the eyes, focal distance through NVGs does not automatically adjust to the distance of the object being fixated.

## Experiment 2: Reach and grab

Although the Howard-Dolman apparatus provides a well-controlled environment in which to test binocular contributions to depth perception, these same controls limit the generalizability of results using this paradigm to real-world contexts. Of interest in the present study was how the provision of binocular depth cues facilitates performance on operationally relevant tasks in less constrained, more ecologically valid, environments.

To address this line of inquiry, a reach-and-grab task was developed that depends on an accurate representation of spatial layout. An array of military-relevant and commonplace objects was positioned on a table, and participants were required to move the objects from the table to a box located at the participant’s hip. Objects were ‘cleared’ in sets of four and the time to clear the set and number of errors made (objects knocked over, dropped) were the key dependent measures. Participants were instructed to clear the objects as quickly as possible without making errors. Although a more traditional military task such as field mobility or field observation could have been chosen (see Tombu et al., 2024), these types of tasks fail to adequately isolate depth perception, instead also capturing potential binocular benefits from binocular summation that do not improve depth perception. The goal here was to assess how binocular depth cues through NVGs facilitate performance, hence the choice of tasks.

Parallelling Experiment 1, the task was performed unaided in well-lit conditions, as well as through NVGs under low-light conditions, in both cases monocularly and binocularly. The focal distance of the NVG was also manipulated, with ideal (focused to the table), near (focused to 8 m) and far (focused to infinity) focus distances assessed. A mixed focal distance condition was not examined in Experiment 2. If binocular depth cues enhance depth perception in less constrained, more realistic settings, performance should be better in the binocular conditions. Moreover, if stereoacuity is preserved even when the NVG is defocused, the binocular advantage should persist as focal distance is manipulated.

A ‘free-viewing’ and a ‘fixed-viewing’ version of the task was performed. In the free-viewing version, participants stood in front of the table and were free to move about as they performed the task. In the fixed-viewing version they sat in a chair at the table with their head in a chin rest. In the former case, additional depth cues were available from self-motion, whereas in the latter, these cues were unavailable. The two versions of the task allowed the contribution from self-motion to be separated out. If cues from self-motion are sufficient to make up for a lack of binocular cues, a reduced or eliminated binocular advantage might be expected in the free-viewing version of the task, but not in the fixed-viewing version. Only a subset of viewing conditions was assessed in the fixed-viewing version.

### Methods

#### Participants

Twenty-four CAF members from 4 Division took part in Experiment 2. Twenty were Regular Force members, and four were members of the Reserve Force. Twenty were men and four were women. Two officers, one senior NCM and 21 junior NCMs acted as participants. Twenty participants were infantry qualified, three were artillery qualified, and one was a combat engineer. In terms of military experience, six participants had less than 2 years, 12 had between 2 and 5 years, four had between 5 and 8 years, one had between 8 and 11 years and one had more than 11 years of experience. Four participants had completed at least one overseas deployment. The average age of participants was 25 years 6 months (*SE* = 10 months). All participants were run during the winter of 2023 (February–March).

All but one participant reported previous field experience with NVGs as part of their military training. Only one participant reported previous experience with a binocular NVG, which they had personally purchased.

Two participants wore glasses or contacts to correct visual acuity to normal. Visual acuity was confirmed binocularly using a Stereo Optical (Chicago, IL, USA) Optec 5500 vision screener.^5^ Mean visual acuity was 0.02 (logMAR, *SE* = 0.014, Snellen equivalent 20/20.66). Three participants were left-handed and 21 were right-handed.

Visual acuity under NVGs was assessed using both the EDTRS charts and the ANV-20/20. EDTRS testing was conducted from 4 m under the lighting conditions used at test with the NVG properly fitted and focused. Mean binocular acuity was 0.32 (logMAR, *SE* = 0.017; Snellen equivalent = 20/41.55) and mean monocular acuity was 0.34 (logMAR, *SE* = 0.017; Snellen equivalent = 20/43.42). Using the ANV-20/20 under ¼-moon conditions, binocular acuity was 0.22 (logMAR, *SE* = 0.016; Snellen equivalent = 20/33.03) and monocular acuity was 0.23 (logMAR, *SE* = 0.013; Snellen equivalent = 20/34.22. Although visual acuity tended to be better under binocular conditions for both tests, in neither case was the difference statistically significant, both *t* values < 1.32, *p* values > 0.19. However, when averaged across the two tests, performance was significantly better in the binocular condition, *t*(23) = 2.25,* p* < 0.04. Consistent with past work (Laird, 1924; Shaad, 1935), the relatively brighter conditions of test in Experiment 2 relative to Experiment 1 may explain the reduced binocular advantage observed in Experiment 2.

#### Testing room and equipment

Testing was conducted in a 3.63-m wide by 6.58-m long room, with off white walls, a white drop ceiling and gray wall-to-wall carpeting. The only door was located on one of the short walls.

As in Experiment 1, a Raid-X was used to provide near infrared lighting, and overhead lights were used to provide normal room lighting. Due to carpeting, the Raid-X was pointed at the ceiling rather than the floor. No room partition was employed, and the lighting was less diffuse than in Experiment 1, producing graded illumination across the room, with objects casting some shadow. Three 6-foot (1.83 m) tables were set up end-to-end lengthwise running from the middle of one of the short walls (the one opposite the door) down the middle of the room. The Raid-X was positioned in the right far corner of the room relative to the door. All fixed-viewing conditions were performed on the far table, closest to the Raid-X, where the same University of Houston College of Optometry Tech chin rest from Experiment 1 was affixed to the left side of the table. Ambient light levels from the Raid-X where the target sets were positioned for the fixed-viewing conditions was 181.7 mlux, as measured by a Hoffman Engineering ANV-410A. The middle table was used for all free-viewing conditions. Near infrared light levels at the free-viewing target array ranged from 72.8 mlux farthest from the Raid-X to 102.5 mlux on the near side. An armless, nonrolling office chair was used to hold the cardboard box (31–cm wide by 31–cm deep by 16.5–cm tall, with foam padding in the bottom) into which participants deposited objects. The third table was used for completing paperwork and for keeping spare objects. A small table in the near right corner of the room was used for the ANV-20/20.

The experimenter sat on the right side of the tables, opposite the participant who was on the left side. The same two EDTRS charts used in Experiment 1 (Charts 1 and 2) were setup on the short wall opposite the door. A tape line was put on the carpet 4 m from the charts. Participants stood at the line to adjust the NVG and to perform vision tests with the EDTRS charts.

A different Thales Bonie LW NVG than that used in Experiment 1 was used in Experiment 2. Helmet and NVG mounts were the same as those used in Experiment 1.

Sixteen objects were used as the items the participants had to remove from the table. See Table 4 for a complete list of the objects, their dimensions and weights. Note that for one participant, the pistol mags were not available for one session and highlighters were used in their place. Objects were chosen to reflect a range of weights, sizes and profiles, and were drawn from military objects (e.g., mags, grenades, glow sticks), as well as more typical everyday items (e.g., batteries, USB sticks, tools) that a soldier might have to interact with.

**Table 4:** List of objects used in Experiment 2 with weight and dimensions

Object	Weight (g)	Width (cm)	Length (cm)	Height (cm)
Pistol mag	88	3	4.5	14
Highlighter*	10	0.7	0.7	12
Water bottle	10	6.5	6.5	20.0
Smoke	380	6.3	6.5	13.5
Frag	414	6.0	6.8	9.0
40 mm	230	4.2	4.2	10.0
C7 mag	434	2.2	8.5	17.3
USB Cable**	26	1.5**	0.8**	83.0**
AA	22	1.4	1.4	5.0
CR123	12	1.6	1.6	3.3
White-out	34	2.3	4.0	6.8
Screwdriver	82	2.6	20.1	2.6
USB stick	4	2.0	5.4	0.7
Bolt & nut	74	1.6	5.7	1.6
Pen	4	1.2	14.5	0.8
Glow stick	20	1.5	1.5	15
Tape roll	150	12.0	12.0	2.4

*Highlighters were used in place of pistol mags for one session for one participant

**USB cable was always balled up. Size varied between the two USB cables used. One was approximately 9 cm × 9 cm × 2 cm, while the other was 12 cm × 8 cm × 2.5 cm

#### Design

The experiment was a fully crossed two-factor within-subjects design, with viewing condition and eye condition as factors, see Table 5. There were six levels of the viewing condition factor: fixed-Unaided (head fixed, seated, natural viewing under typical office lighting conditions), fixed-NVG-table (head fixed, seated, NVG focused to the table), free-Unaided (standing, natural viewing under typical office lighting conditions), free-NVG-table (standing, NVG focused to the table), free-NVG-8 m (standing, NVG focused to 8 m), and free-NVG-Infinity (standing, NVG focused to infinity). There were two levels of the eye condition factor: Monocular (dominant eye) and Binocular.

**Table 5:** Experimental design, Experiment 2

		Eye condition factor	
	Factor levels	Monocular	Binocular
Viewing condition factor	Fixed Unaided	Fixed Unaided Mono	Fixed Unaided Bino
	Fixed NVG Table	Fixed NVG Table Mono	Fixed NVG Table Bino
	Free Unaided	Free Unaided Mono	Free Unaided Bino
	Free NVG Table	Free NVG Table Mono	Free NVG Table Bino
	Free NVG 8 m	Free NVG 8 m Mono	Free NVG 8 m Bino
	Free NVG Infinity	Free NVG Inf Mono	Free NVG Inf Bino

Viewing condition order was counterbalanced across participants with a Williams Design Latin Square. With six viewing conditions a complete counterbalance is achieved every six participants. Four groups of six participants each completed the experiment, for a total of 24 participants. One participant from each group was assigned to each condition order (i.e., each group consisted of a complete counterbalance of conditions order). Monocular and binocular conditions at each level of viewing condition were performed back-to-back. Half of the participants always performed the monocular condition first at each level of viewing condition and the other half always performed the binocular condition first.

Although all conditions were interleaved as part of the counterbalance, fixed- and free-viewing conditions were analyzed independently, as the conditions were not directly comparable due to methodological differences.

#### Procedure

Participants completed Experiment 2 individually across two two-hour sessions on different days of the same week.

The first session began with an overview of the experimental tasks and expectations. The participant then provided informed consent before being fitted with a helmet, NVG mount and NVG, following the procedure described in Experiment 1. Diopter and focus adjustments were then performed to bring the image for each tube into sharp focus, again following the procedure described in Experiment 1. Next, visual acuity through the optimally focused NVG was assessed monocularly and binocularly using the EDTRS charts from 4 m (with chart assignment to eye condition counterbalanced across participants), and with the ANV-20/20 under ¼-moon illumination.

The participant was then given an opportunity to walk around the room and interact with their environment with the NVGs deployed in order to become accustomed to the perspective and image quality provided by the NVG (shifted nodal point, limited field of view, fixed depth of field, etc.).

Once the participant was comfortable with the NVG, practice trials were performed monocularly and binocularly, first unaided under bright lighting conditions (typical office lighting), and then through the NVG, optimally focused to the distance to the table. Practice trials were performed in the ‘free viewing’ condition, with the participant standing in front of the table with two sets of four objects positions on the table. Practice trials, which were intended to familiarize the participant with both the procedure and the actions required to complete the task, followed the same procedure as the experimental trials and are described in detail below. A minimum of two practice trials were completed in each of the binocular and monocular unaided viewing conditions, as well as in the binocular and monocular NVG conditions with the focus distance set to the table (i.e., ideally focused). Once the participant was comfortable with the task, they proceeded to the experimental conditions. Prior to commencing each block of experimental trials, the participant was given the opportunity to move about the room to become accustomed to the NVG configuration being assessed.

**Fig. 3:**
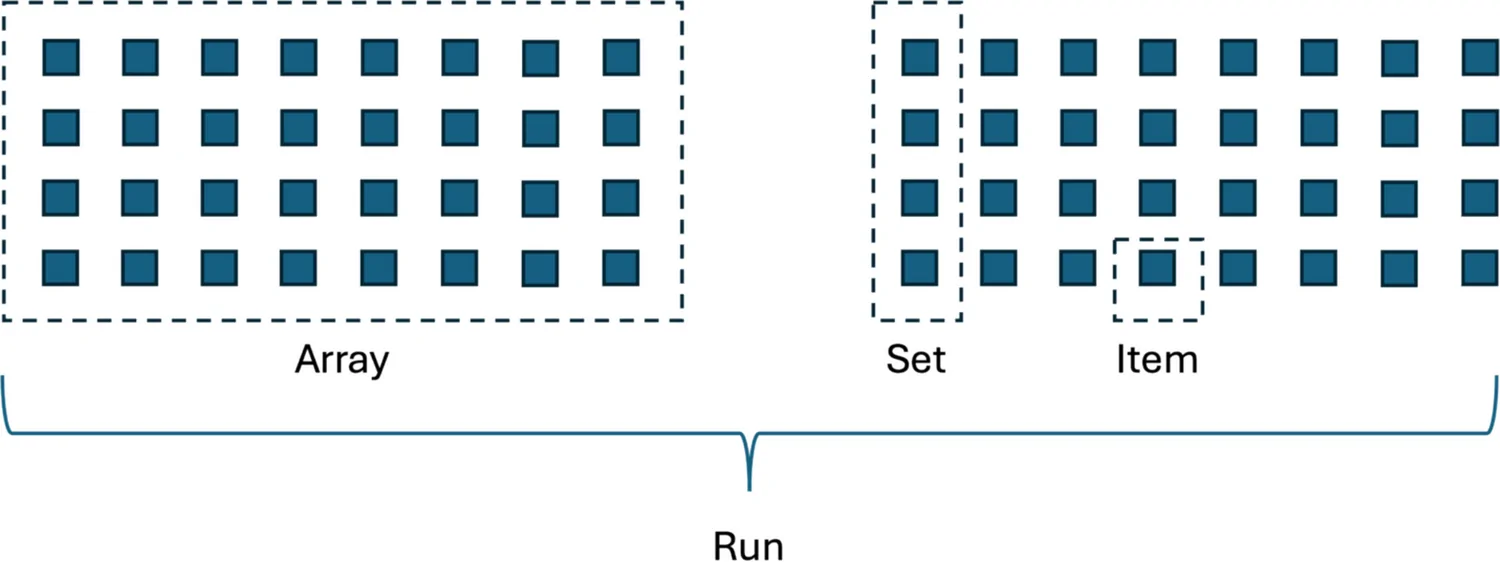
Visual depiction of the stimulus layout used in Experiment 2. On each run, participants cleared two arrays of 32 items each, organized into 16 sets of four items per set. Each participant completed one run in each of the experimental conditions, with the mapping of runs to experimental conditions counterbalanced across participants. On each experimental trial the participant cleared one set of four items in the order indicated by the experimenter. See text for additional details

**Fig. 4:**
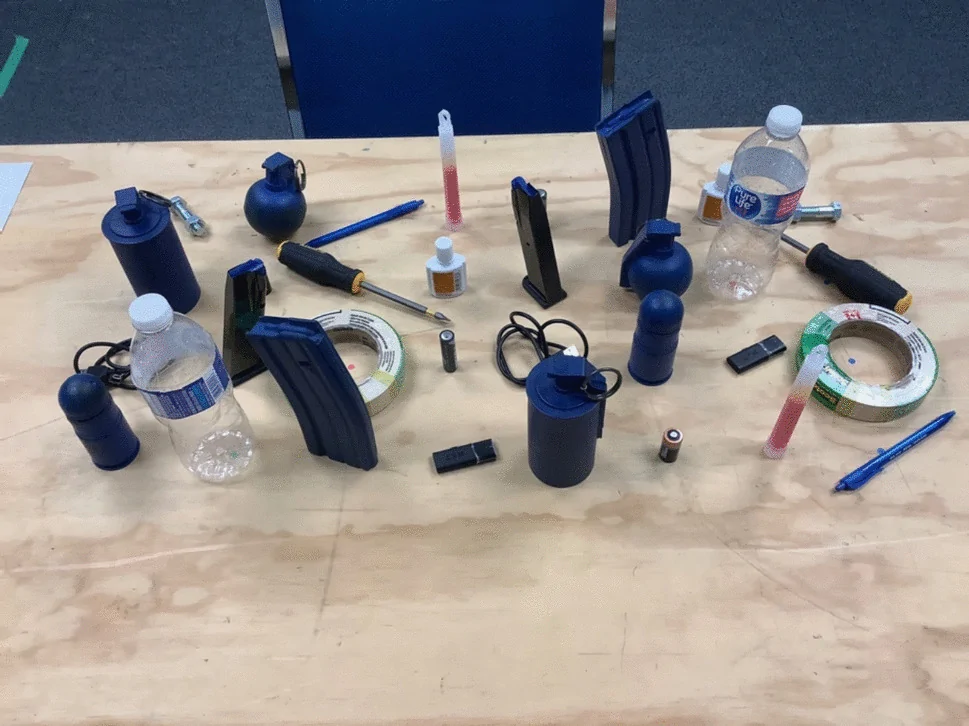
Example of an array of objects used in Experiment 2 under free-viewing conditions from the perspective of the experimenter. The participant would stand on the opposite side of the table (top in picture)

To create the stimuli for the experimental conditions, 24 arrays of 32 items each, consisting of two of each of the objects were created (see Table 4 for a list of the objects; Fig. 3 for a graphical representation of a run, array, set and item; and Fig. 4 for a picture of a free-viewing array). Each array was eight-items wide and four-items deep. Each column in the array (group of four items aligned in depth) is referred to as a ‘set’. Items were assigned to positions in the arrays randomly, with the constraint that identical objects could not appear in the same set. Participants completed two arrays (16 sets) in each run, with one run being completed in each of the experimental conditions. Run 1 always consisted of Arrays 1 and 2, Run 2 always consisted of Arrays 3 and 4, and so on.

On each trial of the experiment, the participant was to remove one set from the table in the order indicated by the experimenter. The item to be removed was identified by number, with ‘1’ indicating the item closest to the participant, ‘2’ indicating the next-nearest item, ‘3’ indicating the item second-farthest from the participant, and ‘4’ indicating the farthest item. Each trial began with the experimenter indicating the first item to be removed. As the participant was removing the just-indicated item, the experimenter would indicate the next item to be removed. This procedure continued until all four items in the set were removed.

Removal orders were determined by randomly ordering the 24 possible item orders and assigning the first 16 orders to sets 1–16, respectively, across the two arrays making up each run. For each run, the same removal order to set mapping was used by all participants (I.e., for set 4 of Run 1, Array 1, all participants first removed the AA battery (item 3), then the USB drive (item 4), then the White-Out bottle (item 2), and finally the glow stick (item 1)).

In the free-viewing conditions, the array (all 32 items) was set up on the middle table, with the first row of items running parallel to and 15 cm from the edge of the table. Item spacing within the array was 10 cm both laterally and in depth.

In the fixed-viewing conditions each set of four items was presented individually in front of the participant, with spacing roughly set to 10 cm between items, but with accommodations being made for participants with shorter arms to ensure that they could comfortably reach the farthest item. The nearest item was always positioned so that it could be seen by the participant while their head was in the chin rest. Items in positions 2–4 were always positioned so that they were clearly visible, with their position being adjusted laterally to avoid occlusion by nearer items.

The 12 runs were always run in the same order, with condition order (and therefore run mapping) counterbalanced across participants. Because condition order was counterbalanced across participants each run was assigned to each condition equally often. This control was put in place to account for set/array difficulty effects which may have resulted in some runs being more difficult than others. Small deviations from the counterbalancing plan resulted from mistakes following the counterbalance. Out of 288 runs completed across participants, 18 were run on the wrong list. Fourteen of these were caused by running viewing conditions in the wrong order, two were caused by running the eye condition for a viewing condition in the wrong order and two resulted from switching lists for two conditions.

For free runs, participants stood in front of the table and aligned themselves with the first set of four items in the array (leftmost set for lefthanded participants, rightmost set for righthanded participants) with the box into which the items were to be deposited positioned on a chair just touching their leg on their dominant hand side. The task was performed with their dominant hand only.

Each trial began with the experimenter, who was positioned on the other side of the table asking if the participant was ready. When they indicated they were, the experimenter would call out the first item to be removed using its corresponding number (e.g., ‘two’), while at the same time starting a stopwatch. The participant would reach for and grab the item, then deposit it into the box. While the participant was removing the first item, the experimenter would indicate the next item to be removed (e.g., ‘four’), and the participant would proceed to remove this item. This procedure continued until all four items were removed. As the fourth item was deposited into the box, the experimenter would stop the stopwatch marking the end of the trial. The experimenter would then record the time to complete the set, and any errors that occurred. Errors consisted of knocking over an item from the set or from an adjacent set or accidentally dropping an item (items that were intentionally dropped into the box were not counted as errors, even if they missed the box, or bounced out). The next trial would then begin, with the participant shifting over to align themselves and the chair with the box with the next set. This procedure was continued until all eight sets making up the array had been removed. The experimenter then turned the lights back on and set up the second array of the run and the procedure was repeated.

For the fixed runs, the same procedure was followed. The only difference being that the participant completed the task from a seated position with their head in the chin rest, and that the experimenter placed each set into position before each trial.

#### Analysis

The primary dependent measures for the reach-and-grab task were time to complete and accuracy. For each participant in each condition, the mean time to complete from all error-free trials was calculated. Accuracy was calculated for each participant in each condition as the proportion of trials where no errors were made.

Fixed-viewing and free-viewing conditions were analyzed independently. Means from the fixed-viewing conditions were subjected to a within-subjects 2 × 2 ANOVA, with viewing condition (Unaided, NVG Table) and eye condition (Monocular, Binocular) as factors.

Likewise, means from the free-viewing conditions were subjected to a within-subjects 4 × 2 ANOVA, with viewing condition (Unaided, NVG Table, NVG 8 m, NVG Infinity) and eye condition (Monocular, Binocular) as factors. Two follow-up ANOVAs were performed, one isolating the impact of moving to an NVG (Unaided/NVG Table × Monocular/Binocular), and the other examining the impact of defocusing the NVG (NVG Table/8 m/Infinity × Monocular/Binocular). Additional follow-up analyses were conducted to isolate differences between conditions as required.

With 24 participants, the design can detect a medium effect size difference (Cohen’s *d* = 0.5) with 0.65 power and a large effect size difference with 0.96 power for both two-tailed matched-sample *t* tests and two-level within-subjects ANOVA main effects. For three-level within-subjects ANOVA main effects, power drops to 0.55 and 0.93 for medium and large effect size differences, respectively. For four-level within-subjects ANOVA main effects, power drops to 0.49 and 0.91 for medium and large effect size differences, respectively.

A Greenhouse–-Geisser correction for violations of sphericity that adjusts the degrees of freedom was applied to all ANOVAs. Raw data can be obtained by contacting the author. This experiment was not preregistered.

### Results

#### Fixed viewing

**Fig. 5:**
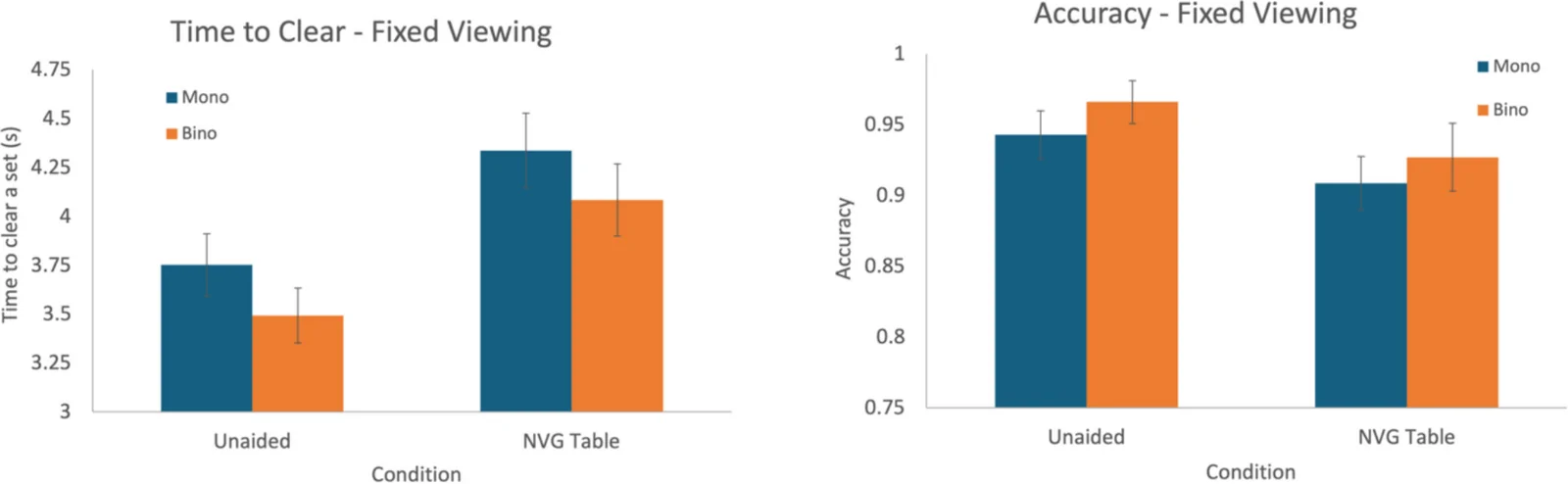
Reach and grab performance under fixed viewing. Testing was conducted Unaided (natural viewing) under well-lit conditions, and though NVGs, ideally focused to the table, under relatively dark conditions (181.7 mlux). In both cases, performance was assessed monocularly and binocularly. Participants were significantly faster to clear sets with unaided viewing and binocularly (left panel). A significant binocular speed advantage was observed for both the Unaided and NVG Table conditions. Participants also made fewer errors with unaided viewing (right panel). Error bars show the standard error of the mean

Mean time to clear a set on error-free trials, and proportion of sets cleared without errors are shown on the left and right of Fig. 5, respectively.

##### Time to complete

A two-factor ANOVA was performed on completion times from fixed-viewing conditions with viewing condition (Unaided, NVG Table) and eye condition (Monocular, Binocular) as factors. Both viewing condition, *F*(1,23) = 21.23, η_p_^2^ = 0.576, *p* < 0.001, and eye condition, *F*(1,23) = 20.87, η_p_^2^ = 0.476, *p* < 0.001, significantly affected performance, with slower completion times observed when the task was performed under NVGs and monocularly. No interaction was detected, *F* < 1. *T* tests confirmed a binocular advantage for both the Unaided and NVG Table viewing conditions, both *t*(23) values > 3.92, *p* values < 0.004, two-tailed.

##### Accuracy

Proportion correct data under fixed-viewing conditions were, submitted to a two-factor ANOVA, with viewing condition and eye condition as factors. Only the main effect of viewing condition was significant, *F*(1,23) = 10.06, η_p_^2^ = 0.304, *p* < 0.005. Neither the main effect of eye condition, *F*(1,23) = 2.87, η_p_^2^ = 0.111, *p* > 0.10, nor the interaction between viewing condition and eye condition, *F* < 1, were found to be significant.

#### Free viewing

**Fig. 6:**
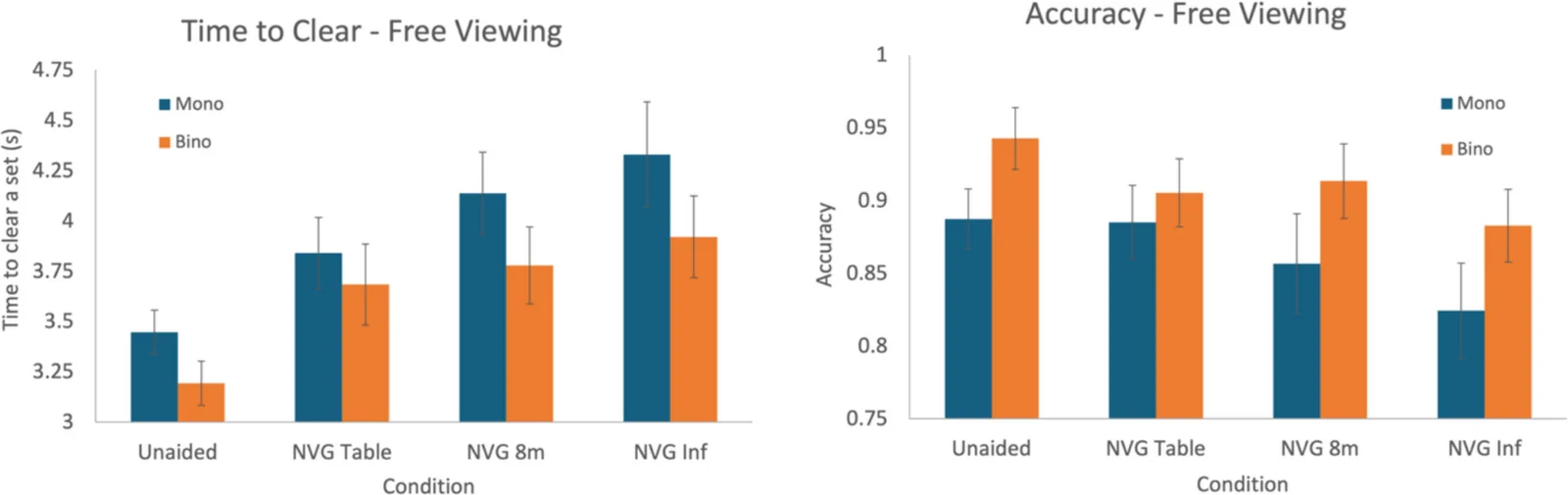
Reach and grab performance under free viewing. Testing was conducted Unaided (natural viewing) under well-lit conditions, and though NVGs at three focus distances (table, 8 m, and infinity), under relatively dark conditions (72.8–102.5 mlux). In all cases, performance was assessed monocularly and binocularly. As viewing conditions degraded, performance degraded for both time to clear a set (left panel) and accuracy (right panel). A significant binocular advantage was also found for both time to clear a set and accuracy. Follow-up tests confirmed the binocular advantage for time to clear a set for all viewing conditions and for accuracy for all viewing conditions except NVG Table. Error bars show the standard error of the mean

Mean time to clear a set on error-free trials, and proportion of sets cleared without errors are shown on the left and right of Fig. 6, respectively.

##### Time to complete

The omnibus two-factor ANOVA, with viewing condition and eye condition as factors, revealed main effects of viewing condition, *F*(2.28,52.44) = 13.90, η_p_^2^ = 0.377, *p* < 0.001, and eye condition, *F*(1,23) = 23.62, η_p_^2^ = 0.507, *p* < 0.001, with a marginal interaction between the two factors,* F*(2.14,49.27) = 2.80, η_p_^2^ = 0.108, *p* < 0.07. As viewing conditions worsened, performance declined. Likewise, sets were cleared slower monocularly than binocularly.

Simple effects of eye condition at each level of viewing condition indicated that a binocular advantage was present in all viewing conditions, all *t*(23) values > 2.0, *p* values < 0.05, two-tailed.

With the presence a binocular advantage established for all viewing conditions by the preceding analysis, additional planned analyses were conducted to examine two theoretically important questions of interest. Specifically, an analysis was undertaken to better understand the impact of moving from unaided to NVG viewing, as was a separate analysis looking at the consequences of defocussing the NVG. Because these tests address different theoretical questions of interest, they have been treated as separate families of tests for statistical purposes, and no adjustment to the critical *p* value has been made. Although such an approach may inadvertently increase the risk of false positives (Type I errors), adjusting the critical p-value has the reciprocal effect of increasing the risk of false negatives (Type II errors). Given the different theoretical questions addressed by each analysis, the choice was made to treat them separately statistically.

To determine the impact of moving from unaided to NVG viewing, a two-factor ANOVA with viewing condition (Unaided, NVG Table) and eye condition (Monocular, Binocular) was conducted. Paralleling the results of the omnibus analysis, significant main effects of viewing condition, *F*(1,23) = 9.38, η_p_^2^ = 0.290, *p* < 0.01, and eye condition, *F*(1,23) = 17.23, η_p_^2^ = 0.428, *p* < 0.001, were detected, with no interaction between these two factors, *F*(1,23) = 2.14, η_p_^2^ = 0.085, *p* > 0.15. Completion times were slower under NVGs and when the task was performed monocularly.

To examine the impact of defocusing the NVG, a two-way ANOVA limited to the NVG conditions was performed with viewing condition (NVG Table, NVG 8 m, NVG Infinity) and eye condition (Monocular, Binocular) as factors. Significant main effects were observed for viewing condition, *F*(1.39,31.92) = 3.92, η_p_^2^ = 0.146, *p* < 0.05, and eye condition, *F*(1,23) = 17.61, η_p_^2^ = 0.434,* p* < 0.001, as was a significant interaction between the two factors, *F*(1.96,45.17) = 3.39, η_p_^2^ = 0.128, *p* < 0.05.

Given the presence of a significant interaction, one-way ANOVAs, with viewing condition as the sole factor, were performed at each level of eye condition. In the monocular analysis a main effect of viewing condition was found, *F*(1.99,33.97) = 4.76, η_p_^2^ = 0.171, *p* < 0.03, whereas in the binocular analysis the main effect was not significant, *F(*1.48,33.93) = 2.11, η_p_^2^ = 0.084, *p* > 0.14. Under monocular conditions, defocusing the NVG negatively impacted completion times, whereas binocularly no effect of defocusing was detected.

##### Accuracy

The results of the omnibus viewing condition by eye condition ANOVA revealed a significant main effect of viewing condition, *F*(2.88,66.24) = 4.40, η_p_^2^ = 0.161, *p* < 0.01, and a significant main effect of eye condition, *F*(1,23) = 13.52, η_p_^2^ = 0.370, *p* < 0.001. As viewing conditions degraded, accuracy degraded, and more errors were committed under monocular than binocular conditions. No interaction between viewing condition and eye condition was detected, *F* < 1.

Although no interaction was found, t-tests at each level of viewing condition did not reveal a binocular advantage in all viewing conditions. Whereas a significant binocular advantage was detected in the Unaided, NVG 8 m, and NVG Infinity viewing conditions, all *t*(23) values > 2.19, *p* values < .02, no such advantage was found in the NVG Table viewing condition, *t*(23) = 1.0, *p* > .16.

As with the time to complete data, follow-up analyses were performed to better understand the impact of changes in viewing condition.

To examine the impact of moving to an NVG, a two-factor ANOVA, with viewing condition (Unaided, NVG Table) and eye condition (Monocular, Binocular), was conducted. A significant main effect of eye condition was observed, *F*(1,23) = 6.26, η_p_^2^ = 0.214, *p* < 0.02, but neither the main effect viewing condition, *F*(1,23) = 1.56, η_p_^2^ = 0.064, *p* > 0.22, nor its interaction with eye condition, *F*(1,23) = 2.06, η_p_^2^ = 0.082, *p* > 0.16, approached significance. Although moving to a monocular configuration resulted in significantly more errors, performing the task under NVGs did not negatively impact accuracy.

The consequences on accuracy of defocusing the NVG was assessed with a follow-on two-factor ANOVA, with viewing condition (NVG Table, NVG 8 m, NVG Infinity) and eye condition (Monocular, Binocular) as factors. Results revealed a significant effect of viewing condition, *F*(1.98,45.56) = 3.22, η_p_^2^ = 0.123, *p* = 0.05, and a significant effect of eye condition, *F*(1,23) = 10.29, η_p_^2^ = 0.309,* p* < 0.005. The viewing condition by eye condition interaction was not significant, *F*(1.67,38.50) = 1.24, η_p_^2^ = 0.051, *p* > 0.29. Follow-up *t* test between viewing conditions collapsing across eye condition indicated that significantly more errors were committed in the NVG Infinity condition that in the NVG Table condition, *t*(23) = 2.35, *p* < 0.03. Marginally more errors were made in the NVG Infinity condition compared with the NVG 8 m condition, *t*(23) = 1.82, *p* < .09. Performance did not differ between the NVG Table and NVG 8 m conditions (*t* < 1).

### Discussion

Taken as a whole, the results of Experiment 2 show that the binocular advantage observed in Experiment 1 extends to a more operationally relevant visuomotor task requiring participants to clear items from a table. Moreover, the binocular advantage was present in all viewing conditions. Even under relatively rich free-viewing conditions where additional depth cues were available (i.e., motion parallax), a significant binocular advantage was observed. Likewise, a binocular advantage was obtained when the task was performed through NVGs, whether in focus, or badly out of focus.

Viewing condition did however affect performance. Both the qualitative shift from unaided to NVG viewing and defocusing of the NVG impaired performance. The former effect could be driven by differences in how the world is viewed through NVGs compared with natural vision. NVGs impose relatively severe field of view limitations and shift the nodal point forward by 10–15 cm, either of which could impair visuomotor performance. Alternatively, the decline in visual acuity, reduced contrast, and/or reduced luminance through NVGs may also account for this effect.

Interestingly, NVG defocusing tended to preserve, or even enhance the binocular advantage, rather than curtail it, suggesting that stereopsis is retained even when the visual input is severely degraded through defocusing. Defocusing clearly impaired performance, however. As the image was defocused, times to clear increased and accuracy decreased.

The interaction (for completion times) between the binocular advantage and defocusing may suggest a common locus of effect (additive factors logic; Sternberg, 1969). If this were the case, defocusing may be impairing monocular depth cues, leading to a greater reliance on binocular depth cues. Whatever the locus, the results of Experiment 2 show that even when defocused, binocular NVGs continue to provide effective binocular depth cues that can be leveraged to enhance visuomotor performance.

## General discussion

The results of this study clearly demonstrate a binocular advantage when performing tasks under NVGs. In Experiment 1, preserved stereopsis was demonstrated through NVGs on a Howard-Dolman task, albeit at levels significantly lower than what can be achieved under natural, well-lit viewing conditions. Furthermore, stereopsis was retained even as the image provided by the NVGs was defocused, so long as both eyes were equally affected.

In Experiment 2, it was shown that this binocular advantage under NVGs extends to a more operationally relevant visuomotor task requiring participants to clear items from a table. Again, the binocular advantage persisted as the NVGs were defocused.

### Possible factors affecting stereopsis through NVGs

Although stereopsis was preserved in Experiment 1, it was significantly reduced in strength relative to natural viewing under well-lit conditions. While this result may be indicative of less effective use of binocular depth cues through NVGs, it could also be expected based on changes in the visual stimulus when viewed through NVGs. Rabin (1996) showed that both contrast attenuation and reduced luminance of NVG displays limit the visual acuity that can be achieved through NVGs. Halpern and Blake (1988) showed that with unaided viewing stereoacuity thresholds increase as contrast decreases Mueller and Lloyd (1948) found that decreasing the luminance of the stimulus also increases depth thresholds. Taken together, these results suggest that limitations in the image produced by NVGs with respect to contrast and luminance may account for the diminished stereoacuity found with NVGs. Improvements in the modulation transfer function of the NVG tubes may therefore lead to improved stereoacuity. Alternatively, mismatches between the tubes of the NVG in terms of magnification, image quality, distortion, and/or alignment may also play a role (Harrington et al., 2014).

Another possibility that has been put forward to explain degraded stereoacuity through NVGs is that it is the result of impaired visual acuity through NVGs (Knight et al., 1998). As previously discussed, while consistent with some results examining the relationship between visual acuity and stereoacuity (Donzis et al., 1983; Goodwin & Romano, 1985; Westheimer & McKee, 1980) and with the observed decrease in stereoacuity moving from well-lit natural viewing to NVG viewing in the present study, it is not supported by the results of the defocusing manipulation in Experiment 1. Had changes in visual acuity been responsible for the attenuated stereoacuity, the defocusing manipulation should have further diminished, or more likely eliminated stereoacuity. In contrast to this expected pattern of results, the defocusing manipulation had no effect on stereoacuity.

While not consistent with some past results (Donzis et al., 1983; Goodwin & Romano, 1985; Levy & Glick, 1974; Westheimer & McKee, 1980), the results of the defocusing manipulation in Experiment 1 are consistent with Stigmar (1971) who found that stimulus blur only affected stereoacuity at the most extreme levels tested. Stigmar introduced blur by placing a semi-translucent screen between the observer and the visual stimulus presented on an oscilloscope. The stimulus consisted of a central vertical bar of light flanked vertically by two vertical bars (one above, one below). The disparity of the central bar relative to the flanking bars was varied, and depth thresholds were collected. The degree of blurring was manipulated by varying the distance between the screen and the oscilloscope and was expressed as the width of the light distribution of the stimulus bars at half intensity height. Across a wide range of blur (from the unblurred, contour-sharp stimulus with a width at half height of 0.5 arcmin, to blurred stimuli up to a width of 4.4 arcmin at half height) blurring had no effect, with stereoacuity only becoming degraded when blurring widened the distribution of light to 7.6 arcmin at half height.

The reasons why stereoacuity has sometimes been found to be extremely sensitive to image blur (Donzis et al., 1983; Goodwin & Romano, 1985; Levy & Glick, 1974; Westheimer & McKee, 1980), and other times has not (Stigmar, 1971, herein) remain unclear. Regardless of the cause, the results of Experiment 1 show a clear decoupling of visual acuity and stereoacuity through NVGs. Whereas visual acuity was severely impaired as the NVG was defocused, dropping from 20/43 to 20/166, stereoacuity was preserved. Importantly, this result implies that binocular depth cues can still be extracted from elements of the scene off the focal plane of the NVG, at least when the tubes of the NVG are focused to the same distance.

In contrast, and consistent with past findings (Donzis et al., 1983; Goodwin & Romano, 1985; Levy & Glick, 1974; Ong & Burley, 1972; Westheimer & McKee, 1980), employing an NVG with tubes focused to different distances was highly detrimental to stereoacuity, reducing it to monocular levels. Operators should be aware of this loss of stereopsis, especially during movement-intensive activities where depth perception plays an important role. The mixed focal distance condition tested herein employed sharply different focal distances with one eye focused to infinity and the other to 3 m. Future work could examine the degree of focal distance mismatch that can be tolerated, if any, while still preserving stereopsis. Ong and Burley (1972) examined induced anisometropia by adding or subtracting up to a two-diopter lens to the dominant eye of normal participants and assessed stereopsis using a Howard-Dolman apparatus. They found that even the addition or subtraction of a 0.5-diopter lens negatively affected depth thresholds, with the size of the impairment increasing as the strength of the lens increased. This result suggests that even a small difference in focal distance between tubes will have a negative effect on stereoacuity.

### Comparative and summative contributions to binocular performance

Both comparative and summative benefits to binocular performance were found in Experiment 1. Improved visual acuity performance was observed under binocular conditions both for well-lit natural viewing conditions and through NVGs. This binocular advantage likely results from binocular summation (Blake et al., 1981). The resulting improvement in the image produced by binocular NVGs could translate into improved task performance for tasks where visual acuity plays a substantial role, such as in target detection and identification (see Tombu et al., 2025).

Comparative processes, specifically binocular convergence and binocular disparities are more likely to explain the binocular advantage found for stereoacuity. The conditions of test in the Howard-Dolman task aimed to isolate the contribution from binocular depth cues, with binocular convergence and binocular disparities having been shown previously to play an outsized role at the range at which testing took place (Cutting & Vishton, 1995). Improved stereoacuity would be expected to improve performance on tasks where depth perception plays an important role, such as the reach-and-grab task examined in Experiment2, which is exactly what was observed.

It is worth stressing the size of the advantage observed in Experiment 2. Under free viewing with NVGs, participants were on average able to clear a set over 300 ms (8%) faster and the error rate was reduced by almost 1/3 (from over 14% to under 10%). To the extent that these benefits transfer to other operationally relevant tasks that depend on depth perception (e.g., mobility) the benefit conferred by binocular NVGs is substantial.

### Learning effects on performance

Despite almost all participants having some experience with NVGs prior to participating in this study, performing visual and visuomotor tasks (Experiments 1 and 2, respectively) through NVGs would still be a relatively unnatural experience for most. As such, the question of learning effects was raised as part of the review. To address this question, performance was examined as a function of block in both experiments.

For the largely visual task examined in Experiment 1 (the Howard-Dolman task), a one-way ANOVA, with block as the factor, was conducted on depth discrimination thresholds. As condition order was counterbalanced across participants, this analysis isolates the effect of block. Importantly, the ANOVA revealed no effect (*F* < 1), indicating no learning effect (average discrimination threshold ranged from a low of 57.9 arcsec [Block 2], to a high of 83.0 arcsec [Block 5], with an average threshold of 70.3 arcsec across all nine blocks).

**Fig. 7:**
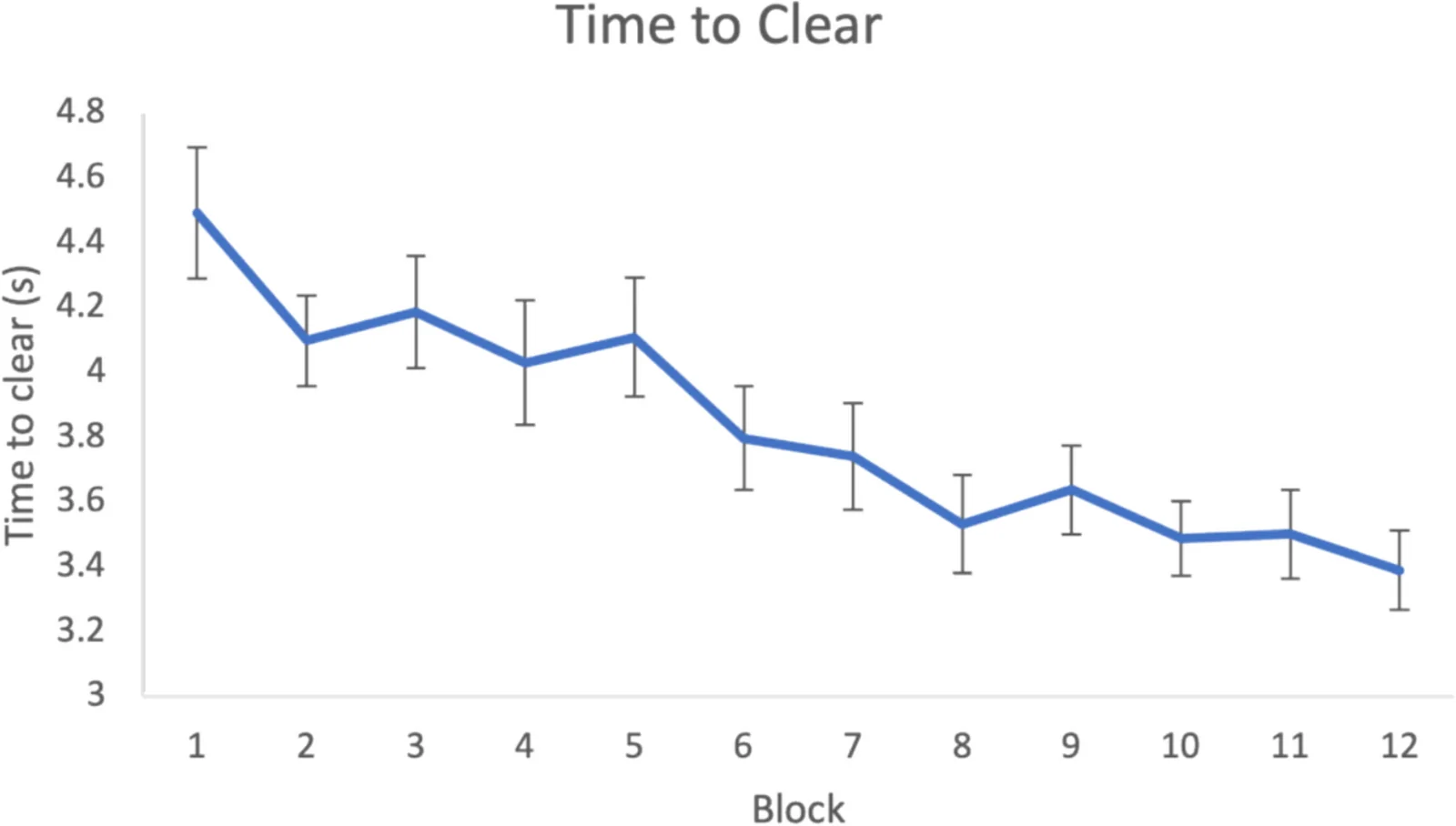
Time required to clear a set as a function of block. The time required to clear a set of four objects decreased almost monotonically as a function of block, appearing to asymptote around Block 8. Importantly, the binocular advantage found over all blocks (282 ms) persisted even for the final two blocks of testing (271-ms binocular advantage). Error bars show the standard error of the mean

In contrast, as shown in Fig. 7 for the visuomotor reach-and-grab task examined in Experiment 2, error-free completion times were affected by block, with performance improving as the experiment progressed, *F*(4.85,111.63) = 11.9, η_p_^2^ = 0.341, *p* < 0.001. Whereas participants took 4.49 s to clear a set in Block 1, only 3.39 s was required to clear a set by Block 12. The same ANOVA conducted on accuracy found no effect of block, *F*(6.20,142.47) = 1.3, η_p_^2^ = 0.052, *p* > 0.27. Because stimulus layout was fixed for each block (i.e., Run 1, which always consisted of Arrays 1 and 2, was always performed in Block 1), block is confounded with stimulus layout. Because the difficulty of clearing a set may be influenced by the specific combination and layout of objects making up a set, and because stimulus layout is confounded with block, it is possible that task difficulty is responsible for the observed effect of block, rather than resulting from a learning effect. Given the generally monotonic decrease in completion times over blocks, however (see Fig. 7), this possibility seems rather remote.

That performance improved over block in Experiment 2 is perhaps unsurprising, as participants became more adept with the task over time. To determine whether learning also influenced the binocular advantage that was observed for all conditions in this experiment (see Results, Experiment 2), the effect of eye condition was examined during Blocks 11 and 12. Although this analysis collapses across viewing conditions, for each participant the same viewing condition was experienced in Blocks 11 and 12—once monocularly and once binocularly, with eye condition order counterbalanced across participants. This analysis revealed a significant effect of eye condition, *t*(23) = 5.3, *p* < 0.001. Importantly the binocular advantage in Blocks 11 and 12 (271 ms) was on par with that observed across all blocks (282 ms over all blocks). This result indicates that the binocular advantage observed in Experiment 2 is not an artifact resulting from a novel task that wears off with experience, but that it persists even as participants become more familiar with the peculiarities of NVGs.

These divergent effects of learning across tasks are interesting. Whereas no learning effects were observed for the largely visual Howard-Dolman task, performance improved over time for the visuomotor reach-and grab task. This result suggests that the visuomotor system takes time to adapt under NVG viewing, whereas no adaptation is required for a purely visual task. Consistent with this interpretation, Gauthier et al. (2008) found that the visuomotor tasks of navigation and way finding under NVGs also improved over time. This divergence between visual and visuomotor performance is likely driven by factors that change moving from natural viewing to NVG viewing and that may be more important in the visuomotor domain than in the purely visual domain. Specifically, restrictions in field of view and nodal point may be more impactful for visuomotor processing than for visual processing. Additional research would be required to fully explore the impact of learning on visual and visuomotor performance.

### Conclusion

Taken as a whole, the results of this study demonstrate the key benefits of binocular NVGs. In line with Knight et al. (1998), binocular NVGs were found to preserve stereopsis for in-focus elements, albeit at a reduced level compared with natural viewing. Moreover, these results extend previous findings to show that stereopsis is retained, even for elements off the focal plane that were significantly out of focus. Furthermore, the binocular advantage extends to an operationally relevant task under realistic conditions where additional cues for perceiving spatial layout are available. This finding shows that the stereoacuity benefit found for the Howard-Dolman task is not simply a laboratory phenomenon that does not extend to real-world situations.

Limitations in depth perception were nonetheless found. Operators should be aware that even under ideal conditions, stereopsis is weaker under NVGs than with natural viewing, possibly stemming from limitations in the quality of the image provided by NVGs (Rabin, 1996). Additionally, stereopsis is not preserved when the focus for each eye is set to a different distance.

Despite the increased cost and weight associated with binocular NVGs, they provide critical benefits that will enhance operator performance on tasks where quickly and accurately perceiving spatial layout are necessary. The ubiquitousness of such tasks on the modern battlefield makes the provision of binocular NVGs essential for personnel tasked with performing such tasks. Future improvements in the modulation transfer function of NVG tubes that enhance image contrast and brightness may further improve the perception of depth that can be achieved through NVGs.

## Data Availability

All materials are described herein. Any additional detail and all raw data can be provided upon request to the author (mike.tombu@forces.gc.ca).

## Footnotes

1 Although past work employing the Howard-Dolman apparatus has typically chosen a 6-m viewing distance, given that binocular depth cues become more powerful at nearer distances (Cutting & Vishton, 1995) a closer distance (3 m) was chosen herein to maximize the benefit provided by binocular viewing.

2 Due to constraints imposed by the length of the room, an 8-m focus distance (the desired near focus distance based on previous work) was not feasible and a 7-m focus distance was instead used.

3 For the first six participants, the experimenter did not block the viewport while resetting the adjustable post. Although these participants were not in the chin rest during this period and indicated that they were not looking while the experimenter adjusted the adjustable post, they recommended that the viewport be blocked so that future participants could not see the adjustment as it was being made. The pattern of results provided by the first six participants were consistent with that provided by the rest of the group and this minor difference in procedure is not expected to significantly affect the results and conclusions drawn from this experiment.

4 To be sure that the choice of baseline (Unaided Monocular) was not obscuring a real binocular advantage in the NVG Mixed condition, comparisons between thresholds from the NVG Mixed and monocular NVG Ideal and monocular NVG Infinity conditions were also performed. These alternative baselines are the constituent focus distances that make up the NVG Mixed condition (one tube set to ideal and the other to infinity). Neither of these tests indicated a binocular advantage either, *t* values(19) < 0.68, *p* values > 0.50.

5 Unlike in Experiment 1, where EDTRS charts were used to assess visual acuity, the maximum visual acuity possible with the Optec 5500 was 20/20. Twenty-two participants had 20/20 visual acuity, one had 20/30 and one had 20/40. The Optec 5500 acuity testing was performed as a screener to confirm normal visual acuity. The upper bound on performance (20/20) made it unsuitable for assessing binocular summation.

## Code availability

No code was produced to execute this study. All analyses were conducted with IBM’s SPSS Statistics for Mac Version 28.0.0.0 (190).
